# Identifying new topoisomerase II poison scaffolds by combining publicly available toxicity data and 2D/3D-based virtual screening

**DOI:** 10.1186/s13321-019-0390-3

**Published:** 2019-11-09

**Authors:** Anna Lovrics, Veronika F. S. Pape, Dániel Szisz, Adrián Kalászi, Petra Heffeter, Csaba Magyar, Gergely Szakács

**Affiliations:** 1 Institute of Enzymology, Research Centre for Natural Sciences, Hungarian Academy of Sciences, Budapest, 1117 Hungary; 2 Present Address: Department of Physiology, Semmelweis University, Faculty of Medicine, Budapest, 1094 Hungary; 3 ChemAxon Ltd., Graphisoft park, Záhony u. 7, Budapest, 1031 Hungary; 4 Institute of Cancer Research and Comprehensive Cancer Center, Medical University of Vienna, Borschkegasse 8a, 1090 Vienna, Austria

**Keywords:** Mitoxantrone, Topoisomerase II poisons, NCI60 cell panel, Virtual screening, Scaffold hopping

## Abstract

Molecular descriptor (2D) and three dimensional (3D) shape based similarity methods are widely used in ligand based virtual drug design. In the present study pairwise structure comparisons among a set of 4858 DTP compounds tested in the NCI60 tumor cell line anticancer drug screen were computed using chemical hashed fingerprints and 3D molecule shapes to calculate 2D and 3D similarities, respectively. Additionally, pairwise biological activity similarities were calculated by correlating the 60 element vectors of pGI50 values corresponding to the cytotoxicity of the compounds across the NCI60 panel. Subsequently, we compared the power of 2D and 3D structural similarity metrics to predict the toxicity pattern of compounds. We found that while the positive predictive value and sensitivity of 3D and molecular descriptor based approaches to predict biological activity are similar, a subset of molecule pairs yielded contradictory results. By simultaneously requiring similarity of biological activities and 3D shapes, and dissimilarity of molecular descriptor based comparisons, we identify pairs of scaffold hopping candidates displaying characteristic core structural changes such as heteroatom/heterocycle change and ring closure. Attempts to discover scaffold hopping candidates of mitoxantrone recovered known Topoisomerase II (Top2) inhibitors, and also predicted new, previously unknown chemotypes possessing in vitro Top2 inhibitory activity.
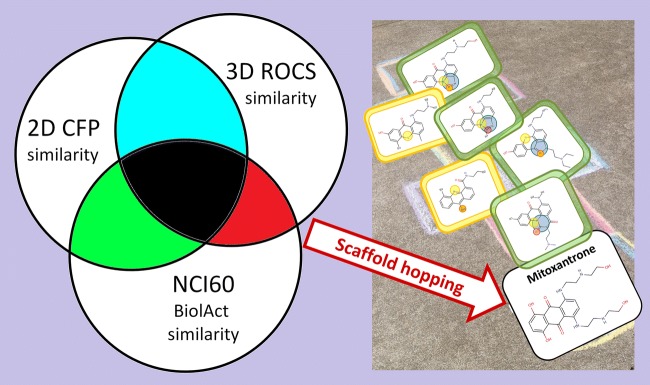

## Introduction

Drug resistance poses a serious challenge in the treatment of malignant diseases or bacterial infections, prompting the need for the development of new drugs. With the increased understanding of the genetic addictions, dependencies and vulnerabilities of cancer cells, target based approaches have yielded several successful treatment options, such as in the case of drugs developed against the epidermal growth factor receptor (reviewed in [1]). In addition, a significant number of novel FDA approved drugs across all therapeutic areas [2] and specifically in cancer [3] have been identified by phenotypic screens.

Target and ligand based approaches are also widely used in virtual drug design. Opposed to target-based design, where drug binding to a known target is tested [4], ligand-based screening can be utilized also when the three dimensional (3D) structure of the target protein is not available [5]. Advances in computational techniques and hardware solutions have enabled in silico methods, in particular virtual screening, to accelerate lead identification and optimization [6].

In phenotypic screens, molecules are characterized by their biological function. The Developmental Therapeutics Program’s (DTP) NCI60 panel is a collection of 60 human cancerous cell lines maintained by the National Cancer Institute (NCI). From 1990 more than 140,000 synthesized compounds and natural products were screened providing a vast repository of molecules for which both toxicity data and structural information are available [7]. Each drug—cell line pair can be characterized by the negative logarithm of the drug concentration that results in 50% growth inhibition of the given cell line (pGI50). Hence, each drug may be described by a 60 element vector, termed ‘biological activity’. Several studies have found that biological activity is a strong predictor of the mechanism of action (MoA) of the compounds [8–13]. Moreover, compounds with previously unknown MoAs were correctly classified (see [7] and references therein), further supporting the use of toxicity-based biological activity patterns as a surrogate for MoA. In addition, by employing molecular descriptor-based methods, where molecules are converted to bit-strings such as chemical hashed fingerprint [14] or extended connectivity fingerprints [15], molecular structures can be analyzed with high speed and at a low computational cost. Methods for comparison of molecule shapes have also been developed to account for spatial features by maximizing the physical overlap of two molecules [16]. Whereas 3D methods have been successfully used to identify chemical leads with different scaffolds [17], 3D screening remains computationally expensive and it is challenging to find the biologically relevant active conformations of the compared molecules.

The relation of molecular descriptors to biological activity of the DTP compounds was extensively analyzed by Wallqvist et al. [18]. Here our aim was to characterize the relationship of different structural similarity measures to the cytotoxic patterns (i.e. biological activity) of the DTP compound set. Interestingly, we identified a set of compound pairs that were dissimilar in molecular descriptor based comparisons, but nevertheless displayed significant biological and 3D shape similarities. The same criteria would also define scaffold hopping pairs representing molecules of different core structures having comparable affinities to their molecular targets [19, 20]. To test this assumption, putative scaffold hopping analogues of the Top2 poison anticancer agent mitoxantrone were analyzed by in silico docking calculations and in vitro decatenation assays.

## Results

### Relation of structural similarity metrics and biological activity of the DTP compounds

In order to assess the relation of structural similarities to biological activity, we calculated pairwise molecular descriptor similarities (chemical hashed fingerprint, CFP), 3D shape similarities (ROCS) and biological activity (BiolAct or BA) similarities among 4858 compounds analyzed by DTP’s NCI60 screening project [7] (see Additional file 1: Fig. S1).

Additional file 1: Fig. S3 shows the distribution of 11,797,653 pairwise similarity values obtained from calculations assessing structural and biological overlaps of the molecules. The pairwise similarity values show a normal distribution, with different means and standard deviations for the structural metrics and the biological activity pattern. We assumed that high values represent significant similarities between the corresponding molecules. Indeed, the right threshold of the 95% confidence intervals (CI) of the bootstrapped distributions representing no-correlation are 0.30 and 0.22 for Pearson-correlation (BA similarity) and CFP similarity, respectively. Bootstrapped distribution could not be obtained for the 3D ROCS method, where the molecules are compared in a pairwise manner (see “Materials and methods”).

In order to test how well either of the structural metrics predict biological activity, the positive predictive value (PPV) and sensitivity were calculated treating the structural metric similarity as the test and Pearson correlation as the true value. In this context, the positive predictive value defines the proportion of molecule pairs that simultaneously display structural and biological activity similarities to the total number of structurally similar molecule pairs (see Eq. (1)). Conversely, sensitivity is the number of molecule pairs that simultaneously display structural and biological activity similarities relative to the number of molecule pairs sharing similar biological activity (Eq. (2)). For any of the metrics, two compounds are considered similar, if their similarity score exceeds a chosen threshold value. Ideally, the selected threshold should warrant not only a high positive predictive value but also a high sensitivity. In our dataset, we find that while an increase of the threshold of a structural similarity metric increases the positive predictive value, it also results in a decrease of sensitivity.

**Fig. 1:**
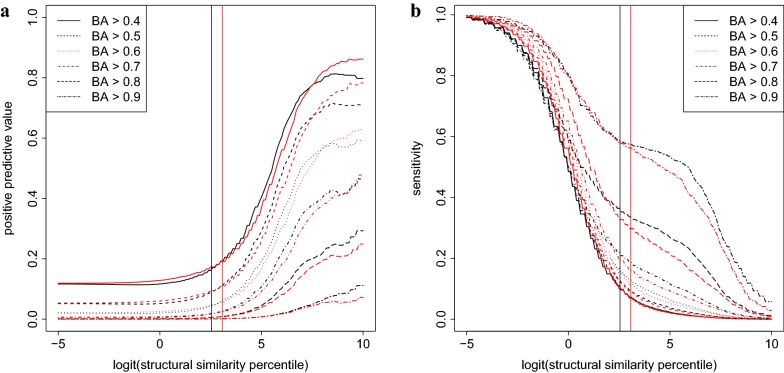
Positive predictive value (**a**) and sensitivity (**b**) of ROCS (black) and CFP (red) similarities to predict biological activity (BA). Values are shown as a function of the percentiles of the number of molecule pairs displaying ROCS or CFP similarities. The thresholds of ROCS and CFP similarity values used for identification of scaffold hopping candidates of mitoxantrone are shown by black and red vertical lines, respectively

**Fig. 2:**
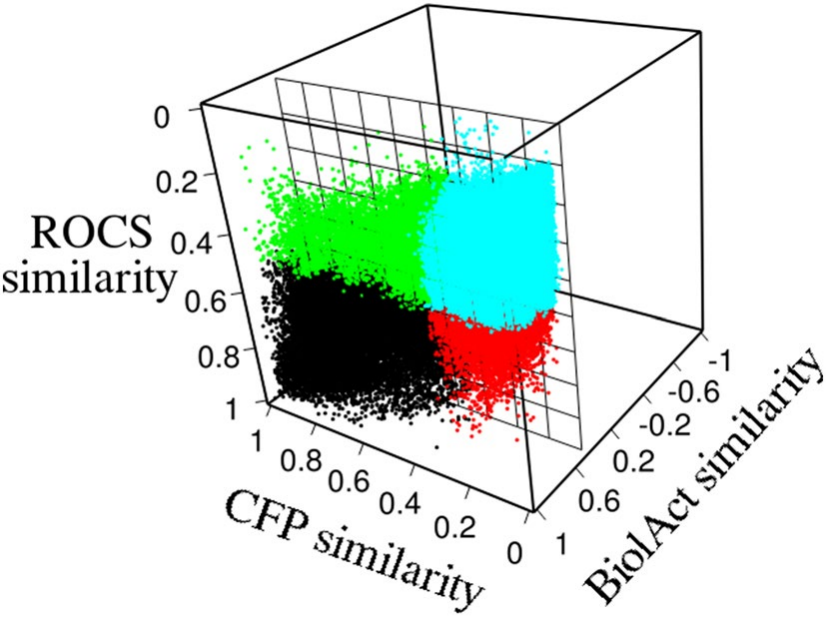
Relation of the three similarity values computed for selected DTP agents. Molecule pairs with high biological activity similarities are shown ($>0.52$). Black: high CFP and ROCS similarities ($>0.34$ and $>0.52$ respectively), green: low ROCS but high CFP similarity, red: high ROCS but low CFP similarity, blue: low ROCS and CFP similarities

Since the distributions of pairwise similarities differ for ROCS and CFP, we introduced percentiles as an independent variable to allow comparison of the 2D and 3D methods. Figure 1 shows that when percentiles are used to define thresholds, the positive predictive value and sensitivity curves are almost superimposable, suggesting that the overall effectivity of the molecular descriptor based and 3D metrics to predict biological activity is highly similar. The percentage of overlap among the distribution curves are 89% and 90% for positive predictive value and sensitivity, respectively. Interestingly, similarity in biological activity and molecular descriptor based structures does not necessarily imply 3D similarity. Likewise, there are molecule pairs that jointly satisfy 3D and biological activity similarities without showing any similarity according to the CFP metric (Fig 2).

We focused on molecule pairs showing high 3D similarity and a highly similar toxicity pattern—yet whose molecular descriptor based similarity did not suggest structural resemblance. This characteristic is reminiscent of ‘scaffold hopping’, i.e. the switch to a new chemotype without a compromise in biological activity.

### Identified scaffold hopping candidates of the Top2 poison mitoxantrone

**Fig. 3:**
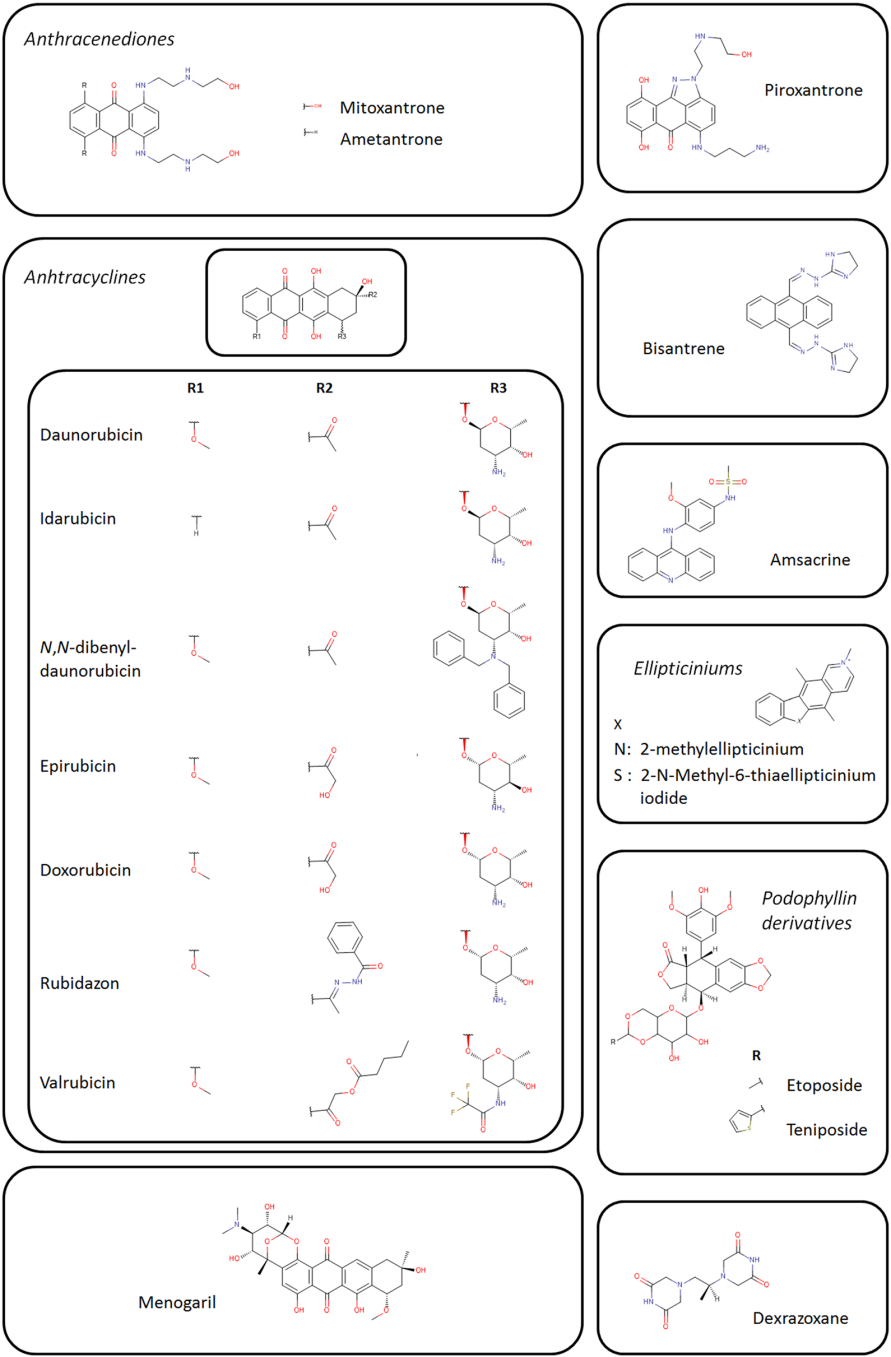
Top2 poisons in the set of 4858 DTP structures used in this study as obtained from Weinstein et al [59], supplemented by mechanism of action (MoA) information downloaded from the CellMiner website. The annotated Top2 poisons include ametantrone (NSC196473/NSC287513), the closest derivative of mitoxantrone, the anthracyclines daunorubicin (NSC83142), idarubicin (NSC256439), *N*,*N*-dibenzyldaunorubicin (NSC268242), epirubicin (NSC256942), doxorubicin (NSC123127), rubidazon (NSC164011) and valrubicin (NSC246131), as well as menogaril (NSC269148). Futhermore, piroxantrone (NSC349174), bisantrene (NSC337766), amsacrine (NSC141549/NSC154948/NSC156303/NSC249992), ellipticiniums (NSC351710, NSC638066) and podophyllin derivatives (etoposide: NSC141540, teniposide: NSC122819), as well as dexrazoxane (NSC169780)

**Fig. 4:**
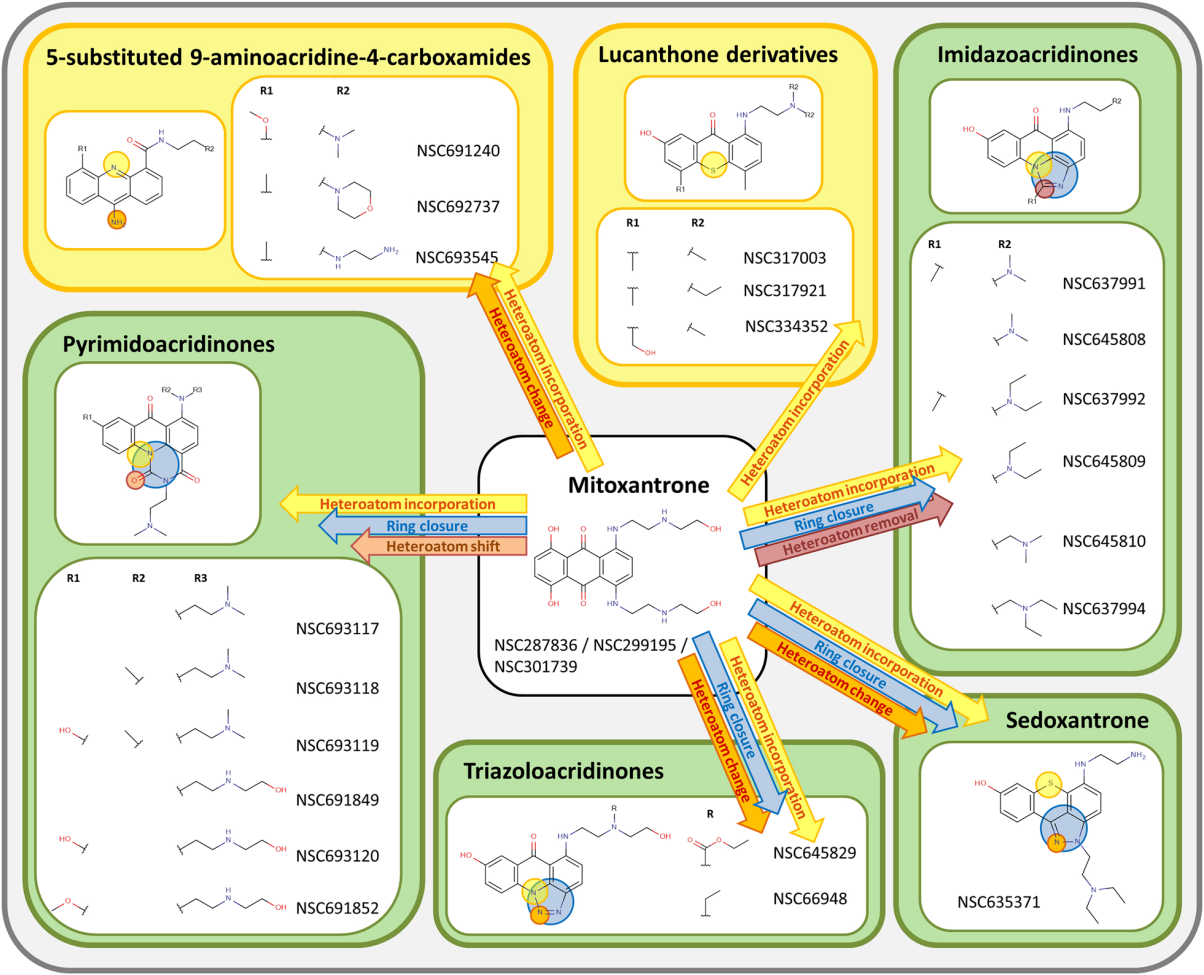
Scaffold hopping candidate molecules around mitoxantrone grouped by their chemotypes. Scaffold hopping candidates are similar to mitoxantrone in their 3D shape, show a similar toxicity pattern in the NCI60 panel and yet can be described by different molecular descriptor-based fingerprints. For each NSC molecule, salts are omitted

In search for scaffold hopping candidates we collected compounds showing high similarity to the 3D structure and the biological activity but low similarity to the molecular descriptor based fingerprint of FDA approved drugs among the 4858 structures analyzed in this study. A prominent group of highly diverse compounds was formed by compounds sharing 3D and biological similarity with the Top2 poison mitoxantrone. Within the subset of these agents, putative scaffold hopping candidates were identified based on dissimilarity of the molecular descriptor-based fingerprints (selection of similarity thresholds is detailed in “Materials and methods”). Briefly, candidates were chosen by considering the similarities of annotated Top2 poisons and inhibitors within the compounds set (Fig. 3) to mitoxantrone. As presented in Fig. 1, the chosen thresholds (BA: 0.53; ROCS: 0.51; CFP: 0.32) represent a compromise between reasonable sensitivity and PPV values for the whole dataset. Within the subset of agents showing high 3D and biological similarity to mitoxantrone ($\text{ BA }\ge 0.54$, $\text{ ROCS }\ge 0.51$), putative scaffold hopping candidates were identified based on dissimilarity of the molecular descriptor-based fingerprints ($\text{ CFP }<0.32$). This approach identified 20 scaffold hopping candidates, representing six distinct chemotypes. As expected based on the similarity criteria, the structure of the compounds show characteristic differences (Fig. 4). SMILES of the scaffold hopping candidates are listed in Additional file S1.

Given the similar biological activity of the compounds shown in Fig. 4, we expected that their MoA relied on Top2 inhibition. Whereas none of the scaffold hopping candidates is annotated as a Top2 poison, some agents, including sedoxantrone (NSC635371), three of the six imidazoacridinone derivatives (NSC637992, NSC645809, NSC645810) [21] and the 5-substituted 9-aminoacridine-4-carboxamides [22] could be linked to Top2 poisoning by literature search. Apart from these compounds, the remaining imidazoacridinone derivatives (NSC637991, NSC637994, NSC645808), the lucanthone derivatives (NSC317003, NSC317921, NSC334352), the triazoloacridinones (NSC645829, NSC699148) and the pyrimidoacridines (NSC693117, NSC693118, NSC693119, NSC693120, NSC691849, NSC691852) represent novel scaffold hopping candidates of mitoxantrone. Based on the range of the pGI50 values measured in the NCI60 cell panel, the activities of these compounds are in the range of the toxicity of mitoxantrone, but their potential to inhibit Top2 has not been investigated so far.

### Verification of the MoA of the scaffold hopping candidates

**Fig. 5:**
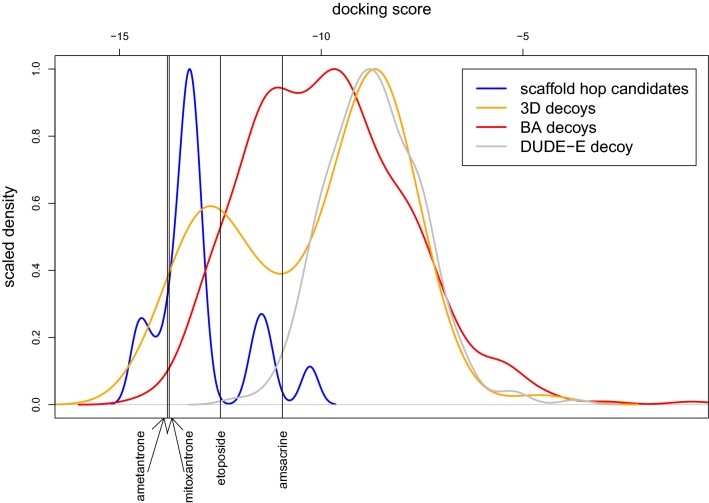
Scaled density of the docking scores calculated for candidate scaffold hopping analogues of mitoxantrone or any of the published Top2 ligands (blue and green, respectively), the ‘3D decoy’ and the ‘biological decoy’ sets (orange and red, respectively) and the DUDE-E decoys (grey). Black vertical lines depict the docking scores of mitoxantrone, ametantrone, amsacrine and etoposide obtained by rigid ligand sampling docking calculations using their own crystal structures

**Fig. 6:**
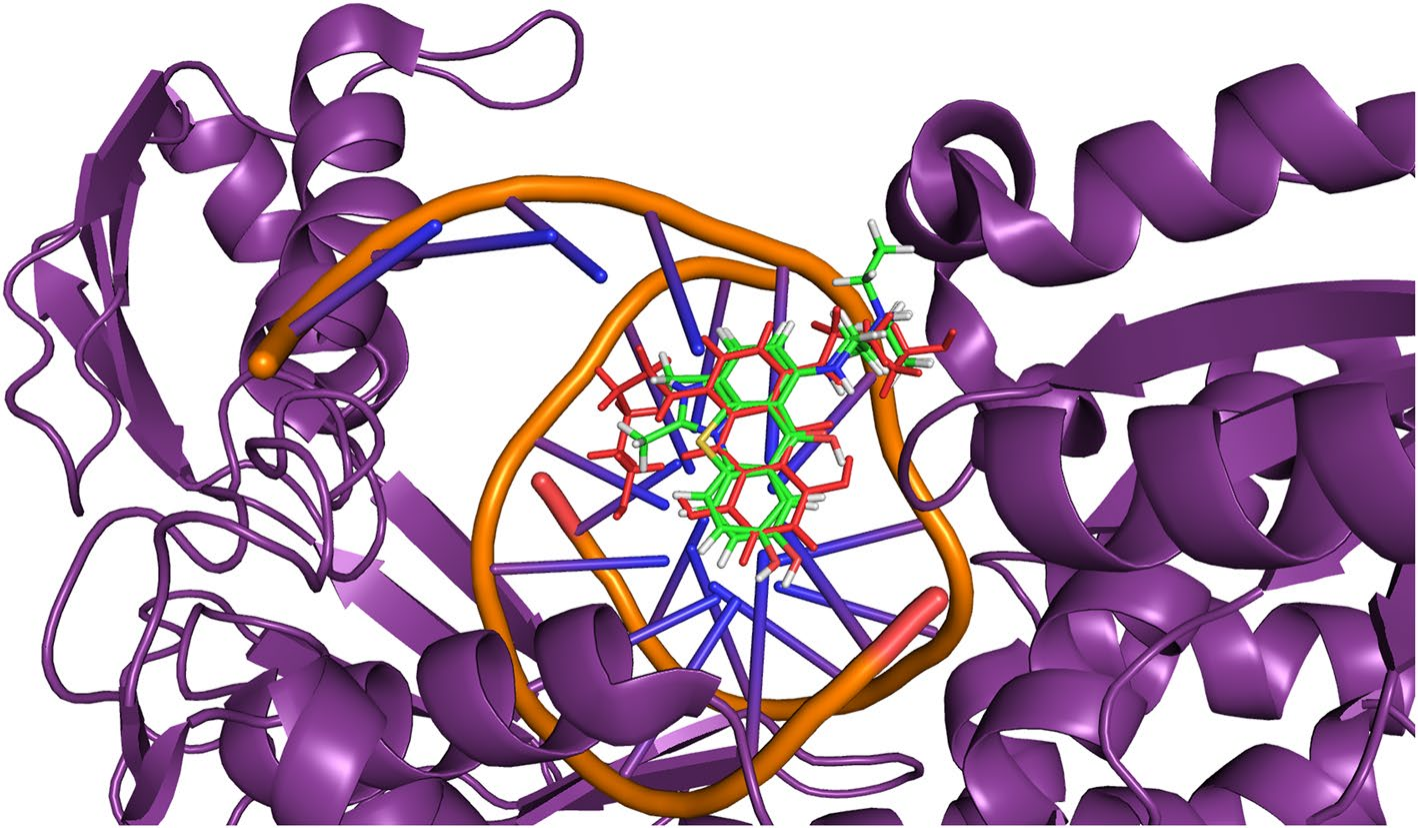
The structure of human Top2${\upbeta }$ is shown with purple color in cartoon, while ligands are shown in stick representation. Co-crystallized structure of mitoxantrone is marked in red color, while poses of NSC317921 and NSC637992 obtained by Glide SP docking calculations are colored using atom types

Similar toxicity patterns (i.e. biological activity) along with similar 3D structures suggest that—similarly to mitoxantrone—the compounds shown in Fig. 4 kill cells by binding to the active site of Top2. To verify this proposition, binding of the scaffold hopping candidates to the Top2-DNA adduct was quantified by in silico docking calculations using protein coordinates reported by Wu et al. [23, 24]. In addition to the scaffold hopping candidates (Fig. 4) and the DUDE-E generated decoy structures, simulations were run for a 3D shape similar but biologically distinct (‘3D decoy’), and a biologically similar but 3D shape distinct (‘biological decoy’) set of molecules. Docking scores obtained for individual molecules are displayed in Additional file 1: Tables S5–S7. Since this search recovered relatively few decoy structures, the search was extended to include similarities in the context of the scores obtained for any of the published Top2 ligands mitoxantrone, ametantrone, amsacrine and etoposide [23, 24]. While five additional putative scaffold hopping candidates arose (Additional file 1: Fig. S4 and Table S3), the overall distribution of the scaffold hopping docking scores did not change (Fig. 5). Docking scores and ranks obtained for the putative scaffold hopping analogues of mitoxantrone are shown in Table 1 and Additional file 1: Table S4, respectively; scores for the ‘3D decoy’ and ‘biological decoy’ sets are shown in Additional file 1: Tables S5, S6. As displayed in Fig. 5, scaffold hopping candidates exhibit a significantly lower docking score than any of the decoy sets, suggesting that the toxic activity of these compounds relies on binding to the Top2-DNA adduct. Mitoxantrone and different chemotypes of scaffold hopping molecules overlap well and share the same binding site, only the longer side-chain of mitoxantrone is involved in additional interactions with the receptor structure (Fig. 6).

**Table 1:** Biological, ROCS and 2D similarities compared to mitoxantrone

Chemotype	NSC	BiolAct	ROCS	CFP	Docking
Mitoxantrone	301739				− 13.45
Lucanthone derivatives	317003	0.73	0.55	0.28	− 11.31
	317921	0.66	0.55	0.29	− 11.47
	334352	0.74	0.55	0.28	− 11.67
Triazoloacridinones	645829	0.56	0.62	0.27	− 13.41
	699148	0.58	0.54	0.26	− 10.28
Pyrimidoacridines	693117	0.67	0.58	0.22	− 13.19
	693118	0.68	0.53	0.22	− 14.48
	693119	0.70	0.57	0.25	− 13.53
	693120	0.70	0.63	0.26	− 14.57
	691849	0.79	0.65	0.24	− 13.81
	691852	0.54	0.63	0.26	− 14.17
Sedoxantrone	635371	0.67	0.60	0.24	− 13.32
Imidazoacridinones	637991	0.60	0.53	0.27	− 13.66
	637992	0.66	0.53	0.28	− 13.25
	637994	0.76	0.52	0.26	− 13.25
	645808	0.76	0.53	0.28	− 13.33
	645809	0.76	0.53	0.29	− 13.00
	645810	0.84	0.52	0.26	− 13.27
5-Substituted-9-aminoacridine	691240	0.68	0.52	0.26	− 13.08
4 Carboxamides	693545	0.63	0.52	0.25	− 12.95

Docking scores of scaffold hopping candidates

**Fig. 7:**
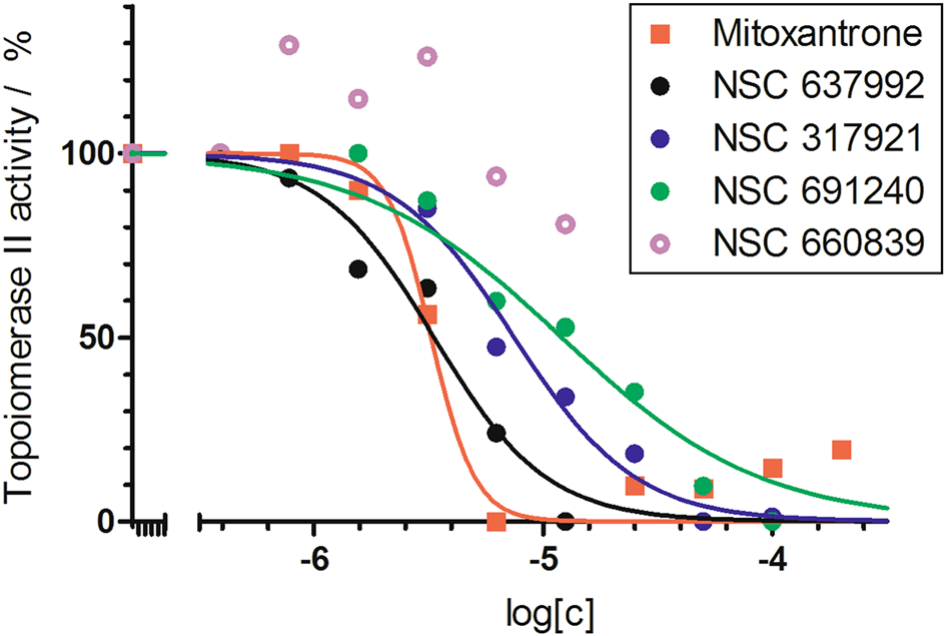
Effect of the test compounds on Top2-mediated decatenation of kinetoplast DNA (KDNA). Dose-dependent poisoning was calculated from DNA samples separated by gel electrophoresis. Symbols represent experimental data, continuous curves are fitted lines. Red: mitoxantrone, black: NSC637992, blue: NSC317921, green: NSC691240, purple: NSC660839. Filled and open circles represent scaffold hopping and decoy molecules, respectively. A representative gel photo is shown in Additional file 1: Fig S5

Finally, we tested the actual Top2 poisoning potential of mitoxantrone analogs and a ‘3D decoy’ (Additional file 1: Fig S7) molecule that were made available by DTP in in vitro decatenation assays. The obtained IC50 value of mitoxantrone is in line with the published literature [25], while for imidazoacridinones, only yeast Top2 assay results were previously available [21]. As shown in Fig 7, the three scaffold hopping analogs representing the imidazoacridinones, lucanthones and the aminoacridines exhibited significant Top2 inhibitory activities (summarized in Table 2), while NSC660839 showed no inhibition, despite the fact, that its docking score (depicted in table S5) was comparable to that of etoposide, a ligand co-crystallized with Top2 [23].

**Table 2:** Calculated Top2 poisoning IC50 values of test compounds

NSC	IC50 (μM)	95% CI (μM)
Mitoxantrone	3.3	2.6–4.1
637992	3.3	2.5–4.6
317921	7.5	5.9–9.6
691240	12.1	9.0–16.3
660839	$>100$	NA

## Discussion

The DTP’s NCI60 is an information rich resource which has provided valuable insights into the MoA of the compounds as well as mechanisms of cellular sensitivity and resistance [7]. Earlier studies have established the relation of drug toxicity profiles to structural clusters and distinct modes of action (reviewed in [7]). In particular, molecular descriptor-based (2D) structural similarity was extensively studied by Wallqvist et al., who quantified the effect of structural changes on biological activity within the DTP molecule set [18]. Our first goal was to compare the power of 2D and 3D structural similarity metrics to predict biological similarity. We used the measures of positive predictive power and sensitivity to assess whether 3D shape based similarities would perform better than molecular descriptor based methods at predicting the similarities of biological activities. In virtual screening campaigns, the positive predictive value can be interpreted as the fraction of truly biologically similar molecules among the compounds obtained by structure similarity search, whereas sensitivity defines the fraction of compounds returned by the structure search among all of the biologically similar molecules. The 2D CFP and 3D ROCS metrics were similar in their ability to predict biological function (Fig. 1), a result in accordance with findings of benchmark studies that have not detected clear evidence of superiority of 3D methods [26–29].

Figure 2 shows the relation of structural and biological similarity metrics. As expected, a large number of molecules are similar in structural and biological aspects. More interestingly, we identified biologically similar molecule pairs for which only either of the 2D or the 3D structural metrics proved to be similar. For example, we found compound pairs that are dissimilar according to the 3D similarity measure, but nevertheless show high similarity in their 2D structures and biological activities (Fig. 2, green). This can occur if a relatively large structural moiety appears once in one of the molecules and multiple times in the other molecule. Naturally, in this case, 3D shape similarity is not observed. On the other hand, such molecule pairs may highlight substructures that are essential for biological activity. Molecule pairs that are structurally dissimilar based on both 3D and 2D comparisons despite their biological activity similarities are displayed in blue. Structural dissimilarity despite biological similarity is a common phenomenon, examples include structurally diverse substrates of transporters [30]; range of ligands of the same protein target that may adopt multiple conformations or simply ligands of different protein targets that belong to the same pathway. Finally, there are agent pairs that are dissimilar in 2D despite high 3D and biological similarities. We hypothesized that this subset would be enriched in scaffold hopping candidates.

An important goal in the initial phase of drug discovery is to increase the quality of drug candidates [31]. Scaffold or lead hopping, defined by similar biological activity of different molecular backbones, could contribute to this aim. Despite their different core structures, scaffold hopping molecule pairs show comparable affinities to their molecular targets [19]. Scaffold hopping analogues may exhibit better physicochemical and pharmacokinetic properties while retaining the original potency, thus providing a new direction for further optimization. Scaffold hopping has been employed to discover novel compounds for drug development in the case of a variety of diseases, including finding scaffold hopping analogues of natural compounds [32]. Overall, finding scaffold hopping variants of active molecules is an integral part of virtual screening in the drug discovery pipeline [5]. While a wide variety of similarity search approaches exist to identify structural analogues to a lead compound, to our best knowledge, there is not a single, commonly accepted in silico method to identify scaffold hopping molecules. Here, we propose a method to identify biologically similar molecules to a query compound that are distinct in their core structure. While an experienced medicinal chemist may readily identify the remote similarity to mitoxantrone of the structures displayed in Fig. 7, it would be impossible to visually screen thousands of compounds. Also, the MoA of these compounds may differ from that of the original active molecule. We introduce similarity of biological activity as an additional criterion to obtain molecule pairs whose MoA is expected to be identical despite differences in their scaffolds.

We tested the validity of our approach by performing in silico and in vitro experiments with scaffold hopping candidates of the Top2 inhibitor mitoxantrone. Since we introduced 2D dissimilarity to mitoxantrone as a condition, the scaffold hopping candidates listed in Table 4 represent different chemotypes. Next to known Top2 poisons, including anthracyclines, structures were recovered that were linked to Top2 poisoning in the literature [21, 22], but some of the analogs represented novel chemotypes for Top2 poisoning. In comparison to mitoxantrone, these structures have additional heteroatoms incorporated in their rings, as in the case of lucanthone derivatives; or a heteroatom incorporation with an additional heteroatom change, as in the case of the 5-substituted-9-aminoacridine 4 carboxamides. A further ring closure can be observed in the triazoloacridinones and in sedoxantrone, which show high similarity to the known Top2 poison piroxantrone (see Fig. 3). Imidazoacridinones are derivatives of mitoxantrone, in which a heteratom is incorporated into a ring, another one is removed and additionally a ring is formed. Pyrimidoacridinones differ from mitoxantrone by a heteroatom incorporation, a ring closure and a shift of another heteroatom. A remote similarity of the scaffold hopping candidates can also be seen to amsacrine, which is a known Top2 poison (Fig. 3). However, these molecules still represent new chemotypes among Top2 poisons.

As the principal MoA of mitoxantrone is Top2 poisoning, activity of the scaffold hopping set could be verified by in silico docking and in vitro decatenation assays.

Scaffold hopping candidates of mitoxantrone obtained similar docking scores as the published Top2 ligands mitoxantrone and ametantrone, and notably, better docking scores than etoposide and amsacrine and the majority of known Top2 poisons. By defining biological and structural decoy sets (agents where only either the biological activity or 3D structural similarity to a published Top2 ligand is above the chosen thresholds), we found that both properties were required to obtain good docking scores. We note that some of the biological decoy molecules possess docking scores as good as the scaffold hopping candidates, and hence can be thought of false negatives. However, on the one hand, it is expected that some structurally diverse molecules would also fit well to the Top2 binding site. Still, the majority of structurally distinct biological decoys display a worse docking score. Taken together, some scaffold hopping candidates might have been lost by applying the methodology presented in this paper, but the number of false positives was also reduced significantly. On the other hand, ROCS similarity was found to perform better than docking in search for active molecules [5], and hence it could also be hypothesized, that in some cases, the docking calculation produces false positive results.

In order to test whether using different thresholds for the similarity metrics would provide better docking score separation between the scaffold hopping candidates and the biological decoy molecules, Fig. 5 was replotted using stricter (higher BiolAct and ROCS and lower CFP) and more lenient (lower BiolAct and ROCS and higher CFP) similarity threshold values. While at more stringent threshold values the separation of scaffold hopping candidates and decoy molecules is more pronounced, there remains a subset of ’3D decoy’ molecules displaying good docking scores (Additional file 1: Fig. S6).

Scaffold hopping candidates made available by DTP were also evaluated in in vitro decatenation assays, which demonstrated that NSC637992, NSC317921 and NSC691240 are efficient Top2 poisons, while NSC660839, a ’3D decoy’ molecule does not show inhibitory power despite the fact that it obtained a good docking score.

The list of 4858 DTP compounds used in this study and their pairwise structural and biological similarities are available in Additional files S2 and S3 respectively. Based on this dataset, scaffold-hopping candidates of any arbitrary agent may be obtained. As a further example, scaffold hopping candidates of camptothecin (NSC94600), a Topoisomerase I (Top1) inhibitor were also collected [33]. Additional file 1: Figs. S8 and S9 depict known Top1 inhibitor scaffolds and the scaffold hopping candidates, respectively. Threshold values to obtain scaffold hopping analogues around camptothecin were obtained similarly to those for mitoxantrone (see “Materials and methods”), by comparing the biological activity to known Top1 inhibitors. However, this approach could not be followed to identify scaffold hopping analogues of podofilox (NSC24818). Podofilox targets Eg5, a human kinesin involved in the formation of the bipolar spindle [34], and as such, could be categorised as tubulin affecting antimitotic. Still, biological activities of DTP compounds annotated as tubulin affecting agents display such a wide variety, that the minimum of the biological activity similarities of these molecules to podofilox represent random correlation among the set of 4858 DTP agents. Hence, in the case of podofilox, putative scaffold hopping candidates were selected using similarity thresholds corresponding to the 90th percentile of all pairwise similarities for each metric. Additional file 1: Figs. S10 and S11 represent the structure of podofilox and its putative scaffold hopping analogs, respectively.

The presented methodology can be applied to any set of molecules whose biological activity can be quantitatively compared in a pairwise manner. For this purpose, the DTP NCI60 database serves as a unique resource, but CMap [35], SIDER [36, 37] or chemogenomic databases [38] or the calculated ADMET properties [39] could also provide the starting point to search for candidate scaffold hopping molecule pairs. Similarly, different structural similarity metrics could be employed. For the molecular descriptor similarity calculations, ChemAxon’s CFP [40] was chosen as a well-established, widely used measure, however other similar metrics (e.g. the Extended Connectivity Fingerprint) may also be applied. OpenEye’s ROCS was chosen for 3D calculations as the most widely used and tested method [17], however, other alternatives exist (e.g. ChemAxon’s Screen3D [41], pharmacophore fingerprints using either fuzzy molecular representations [42] or combined with ranking, voting, and consensus scoring [43]). It has to be noted that parametrization of the chosen metric, the similarity calculation method and the selection of the thresholds may also influence the outcome of similarity estimations. For instance, in the ROCS calculations, the most similar conformations among tested molecule pairs were used to assign the similarity score of the compounds. If the biologically active conformations are not known or in case of a general database search this might be the method of choice, however, the presented in silico application to find scaffold hopping analogues of a main compound should always be tailored to the actual task.

## Conclusion

We have introduced a method to generate scaffold hopping molecule pair candidates by simultaneously calculating biological activity, 3D shape and molecular descriptor based similarities. Scaffold hopping candidates of mitoxantrone displayed typical examples of core structural changes such as heteroatom/heterocycle change and ring closure. The method was able to recover known Top2 inhibitors and additionally predicted new, previously unknown chemotypes possessing in vitro Top2 inhibitory activity.

## Materials and methods

### Description and curation of DTP toxicity data and molecular structures

Structural information and pGI50 values were downloaded from the DTP websites (https://wiki.nci.nih.gov/display/NCIDTPdata/Chemical+Data and https://wiki.nci.nih.gov/display/NCIDTPdata/NCI-60+Growth+Inhibition+Data: GI50 Data (Sept 2014) respectively). Chemical and biological data curation inspired by [44, 45] is detailed in Additional file 1: Text and Fig. S1.

Briefly, for downloaded agents, pGI50 values were available for some or all of the NCI60 cell lines. Missing values or GI50 values equal to tested minimal or maximal drug concentrations were replaced by ‘NA’. Compounds with more than 30 ‘NA’ values were omitted; the remaining set was filtered to retain compounds showing variable toxicity (standard deviation of pGI50 values $\ge 0.4$) [46, 47]. Correlations of pGI50 values across the cell lines of compounds measured multiple times were generally good as shown in Additional file 1: Fig. S2, demonstrating the reliability of the DTP dataset.

Biological curation was followed by chemical standardization: non-covalently bound fragments were removed from the structures using ChemAxon’s Standardizer [48]; if these fragments were physiologically relevant ions (i.e. Na$^{+}$, Cl$^{-}$, ${\text {SO}_{4}}^{2-}$), then the desalted compound was retained, otherwise the agent was omitted. Additional structures were either fixed when possible, or removed based on problems related to valence, formal charge and stereochemistry as defined by Structure Checker (ChemAxon) [49]. Inorganics and metal-containing molecules were also removed using an in-house script. Remaining agents were dearomatized and nitro groups were transformed into customized representations by ChemAxon’s Standardizer [48]. The final structures were tested by both ChemAxon’s Structure Checker [49] and OpenEye’s OMEGA [50]. Since the DTP structure set only contains 2D structure information, compounds with undefined stereocenters were kept—even though this added some uncertainty to the 3D shape-based similarity calculations.

The final set contained 4858 unique structures after removal of desalted molecules that represented duplicate or triplicate structures as obtained by ChemAxon’s duplicate search [51]. In the case of low biological activity correlation among duplicate structures, the agents were omitted, while in the case of high correlation of toxicity values of the duplicate structures, pGI50 values were averaged resulting in a final set of 4858 unique structures. The workflow of molecule selection is depicted in Additional file 1: Fig. S1.

### Similarity calculations

Pearson correlation was used to calculate the similarity between biological activites (drug toxicity profile vectors) of the DTP compounds, handling missing pGI50 values by casewise detection. In total, 11,797,653 pairwise similarity values were obtained. ROCS (OpenEye Scientific Software, Santa Fe, NM) was used to calculate 3D shape-based overlaps [52, 53]. For calculating 3D similarity, each compound was expanded into a set of 3D conformers using OpenEye’s OMEGA. For each molecule, a maximum number of 200 conformers were generated and assembled in an energy sorted order [50, 54]. When a molecule contained undefined stereocenters, random stereocenters were defined during conformer generation. For each pair of structures, 3D overlaps between all of the the available conformers were calculated using ROCS [52, 53], by applying the ‘-subrocs’ option (starting the search at heavy atoms of the larger molecule) without further optimization. The highest similarity score was accepted as the ‘ROCS’ similarity between the selected pair of molecules. To obtain structural similarity based on molecular descriptors, ChemAxon’s chemical fingerprint (CFP) [40] was utilized using the default parameters and the Tanimoto metric as the similarity measure. Similarity was calculated between each of the 4858 individual structures, resulting in 11,797,653 pairwise similarity values. The exact commands and parameters used for the molecular similarity methods are shown in Additional file 1: Text, and Fig. S1 summarizes the similarity calculations.

Motivated by the work of Wallqvist et al. [18], we define

1 $$\begin{aligned} F(s=s_T| \rho = \rho _T)= & {} \dfrac{N(s \ge s_T; \rho \ge \rho _T)}{N(s \ge s_T; \rho \ge -1)}, \end{aligned}$$

2 $$\begin{aligned} F(\rho = \rho _T| s=s_T)= & {} \dfrac{N(s \ge s_T; \rho \ge \rho _T)}{N(s \ge 0; \rho \ge \rho _T)}, \end{aligned}$$

where $N(s \ge s_T; \rho \ge \rho _T)$ represent the number of molecule pairs for which the value of the structural similarity metric (*s*) is larger or equal than the requested threshold $s_T$ and the value of the Pearson correlation ($\rho$) is simultaneously larger or equal than the requested threshold $\rho _T$.

The measures in Eqs. (1–2) quantify how well structural similarities predict biological response. Specifically, $F(s=s_T| \rho = \rho _T)$ expresses the fraction of molecule pairs with at least $s_T$ structural similarities that also show a minimum of $\rho _T$ biological activity similarities, i.e. the positive predictive value. Similarly, $F(\rho = \rho _T| s=s_T)$ indicates the fraction of molecule pairs with at least $\rho _T$ biological activity similarities that also share a minimum of $s_T$ structural similarities, i.e. the sensitivity. These measures were calculated for both structural metrics (ROCS and CFP) among the selected DTP agents.

### Similarity threshold selection to mitoxantrone

Selected cut offs between agent pairs considered similar or dissimilar can be tailored to the needs of the actual study, considering expected increase and decrease of false positives and negatives. Here, we show a possible procedure to select actual threshold values to differentiate between low and high similarities.

In order to define a suitable Pearson correlation threshold value, the biological activity of mitoxantrone was compared to a set of known Top2 poisons and inhibitors (Fig. 3). The threshold of biological activity similarity was set to be at least 0.44, representing the lowest similarity to mitoxantrone among the annotated Top2 poisons and inhibitors see Additional file 1: Table S1. The procedure to select potential threshold intervals for ROCS and CFP similarities was as follows. First, the percentiles of molecule pairs displaying 0.44 BA similarities were calculated, then structural threshold values corresponding to these percentiles were obtained. For each possible combinations of BA, ROCS and CFP threshold values, the number of putative scaffold hopping molecules was determined. The molecules remain the same when $\text{ BA }>=[0.53,0.54]$, $\text{ ROCS }>=[0.50,0.52]$ and $\text{ CFP }<[0.32,0.34]$, the final threshold values were hence chosen as 0.54, 0.32 and 0.51 for BA, CFP and ROCS similarities respectively (Additional file 1: Table S2).

### In silico docking

Human topoisomerase IIb in complex with DNA and etoposide, mitoxantrone, ametantrone and amsacrine (3QX3, 4G0V, 4G0W and 4G0U in PDB) was analyzed in docking calculations [23, 24, 55], using the Small-Molecule Drug Discovery Suite 2017-1 (Schrödinger, LLC, New York, NY, 2017) [56]. All four protein structures were prepared using the Protein Preparation Wizard, H-bonds were optimized with the automated procedure. A fifth protein structure was also used during docking calculations: a 4G0U—etopiside complex structure resulting from induced fit docking (IFD) calculations. Ligands were prepared by the Ligprep module with default parameters except that the maximum number of stereoisomers was set to 4. For each target structure, the binding site was defined based on the corresponding drug molecule coordinates. For each ligand, the best docking score was used in the follow up analysis. The decoy library was generated using the DUD-E online tool at http://dude.docking.org/generate [57]. Additionally, ‘3D similarity decoys’ were selected from the DTP agents as molecules, whose 3D shape was similar to a published Top2 ligand [23, 24], but their biological activity based Pearson correlation similarities were low. Conversely, ‘Pearson similarity decoys’ were selected, whose 3D shape was dissimilar, but their drug toxicity profile was similar to a published Top2 ligand. Docking calculations were performed on all ligand and decoys structures using the Glide SP method (Schrödinger, LLC, New York, NY, 2017) [56]. Figure 6 was created using the PyMOL program. (The PyMOL Molecular Graphics System, Version 1.6, Schrödinger, LLC.)

### In vitro decatanation assay

The inhibitory effect of the compounds on the catalytic activity of Top2 was investigated using the decatenation assay (TopoGEN, Ohio) [58]. $0.2\, \upmu \text {g}$ catenated kinetoplast DNA (kDNA) was incubated at 37 °C for 30 min in the presence of the test compounds and Top2 in a final volume of $20\,\upmu \text {l}$, containing 50 mM Tris–Cl (pH 8.0), 150 mM NaCl, 10 mM $\text {MgCl}_{2}$, 5 mM ATP, 0.5 mM DTT and $30\,\upmu \text {g}/\text {ml}$ BSA. Mitoxantrone was used as a positive control. The reaction was stopped by a 15 min incubation at 37 °C with $3\,\upmu \text {l}$ SDS containing $1\text { mg}/\text {ml}$ proteinase K. Samples were separated by 1% agarose gel electrophoresis (100 V, 30 min). DNA bands were visualized by ethidium bromide. UV-transilluminated gels were documented with the Multi-Analyst software. Dose response curves were fitted to experimental data using the equation $\hat{y} = b + (t-b) \times \log (\text{ IC50 })^n/(\log (\text{ conc })^n+\log (\text{ IC50 })^n)$, where $b=0$, $t=100$.

## Supplementary information

**Additional file 1: Additional text.** Additional Text includes the commands used for the ROCS (OpenEye Scientific Software, Santa Fe, NM) and CFP (ChemAxon Ltd., Budapest, Hungary) similarity calculations and Additional Figures and Tables. **Figure S1.** Flowchart depicting the selection and comparison of DTP molecules used in this study. **Figure S2.** Histogram of pairwise Pearson correlation values among NSC duplicates (a) and desalted structure duplicates (b). Dashed vertical red line represents the Pearson correlation threshold used in this study to select scaffold-hopping analogues of mitoxantrone, while continuous red vertical line represents the cut-off for keeping duplicate structures. **Figure S3.** Distribution of the 11,797,653 pairwise similarity values supplemented with the bootstrapped distributions (continuous lines), where available. The vertical lines show the 95% confidence intervals of the bootstrapped distributions. **Figure S4.** Additional scaffold hopping candicates of either mitoxantrone, ametantrone, amsacrine or etoposide. Cf. Fig 3 in main text. **Figure S5.** Example gel photos displaying dose-response Top2 poisoning of NSC637992 and mitoxantrone. Dose response curves were calculated based on the intensities corresponding to the decatenated DNA (red arrow). **Figure S6.** Scaled density of the docking scores calculated for candidate scaffold hopping analogues of mitoxantrone (blue), the ‘3D decoy’ and the ‘biological decoy’ sets (orange and red, respectively) and the DUDE-E decoys (grey) when the similarity threshold values were chosen as the strictest (a) and most lenient (b). **Figure S7.** NSC660839, the ‘3D decoy’ molecule tested in the in vitro decatenation assay. **Figure S8.** Known Top1 inhibitor scaffolds: camptothecins (NSC94600), indenoisoquinolines (NSC314622), indolocarbazoles. **Figure S9.** Scaffold hopping candidates of camptothecin obtained using threshold values BA > 0.49, ROCS > 0.51, CFP ≤ 0.34. **Figure S10.** NSC24818 (podofilox). **Figure S11.** Scaffold hopping candidates of NSC24818 obtained using threshold values BA > 0.41, ROCS > 0.48, CFP ≤ 0.30. **Table S1.** Pearson correlation (BiolAct similarity) of the pIC50 values of annotated Top2 poisons to mitoxantrone. **Table S2.** Selected similarity thresholds to identify putative scaffold hopping analogues of mitoxantrone. **Table S3.** Docking scores and rankings (from 1173 compounds) of mitoxantrone and its scaffold hopping candidates. **Table S4.** Maximum of biological, ROCS and 2D similarities compared to mitoxantrone, ametantrone, amsacrine or etoposide. Additionally, docking scores of these compounds. **Table S5.** Similarities compared to published Top2 ligands and docking scores of 3D decoy agents. **Table S6.** Similarities compared to published Top2 ligands and docking scores of biological activity decoy agents. **Table S7.** Docking scores of DUDE-E decoys.

## Data Availability

Additional files S1, S2 and S3 are available at https://drive.google.com/drive/folders/1V8mjE0KNsr0SLI0VaEAqeSgIy_wyHyUL?usp=sharing.
