# Supplementary material for: Identifying new topoisomerase II poison scaffolds by combining publicly available toxicity data and 2D/3D-based virtual screening
Source: J Cheminform. 2019 Nov 9;11:67. doi: 10.1186/s13321-019-0390-3 (PMC6842385; doi:10.1186/s13321-019-0390-3)
Supplement: Supplementary file 1 — Additional file 1: Additional text. Additional Text includes the commands used for the ROCS (OpenEye Scientific Software, Santa Fe, NM) and CFP (ChemAxon Ltd., Budapest, Hungary) similarity calculations and Additional Figures and Tables. Figure S1. Flowchart depicting the selection and comparison of DTP molecules used in this study. Figure S2. Histogram of pairwise Pearson correlation values among NSC duplicates (a) and desalted structure duplicates (b). Dashed vertical red line represents the Pearson correlation threshold used in this study to select scaffold-hopping analogues of mitoxantrone, while continuous red vertical line represents the cut-off for keeping duplicate structures. Figure S3. Distribution of the 11,797,653 pairwise similarity values supplemented with the bootstrapped distributions (continuous lines), where available. The vertical lines show the 95% confidence intervals of the bootstrapped distributions. Figure S4. Additional scaffold hopping candicates of either mitoxantrone, ametantrone, amsacrine or etoposide. Cf. Fig 3 in main text. Figure S5. Example gel photos displaying dose-response Top2 poisoning of NSC637992 and mitoxantrone. Dose response curves were calculated based on the intensities corresponding to the decatenated DNA (red arrow). Figure S6. Scaled density of the docking scores calculated for candidate scaffold hopping analogues of mitoxantrone (blue), the ‘3D decoy’ and the ‘biological decoy’ sets (orange and red, respectively) and the DUDE-E decoys (grey) when the similarity threshold values were chosen as the strictest (a) and most lenient (b). Figure S7. NSC660839, the ‘3D decoy’ molecule tested in the in vitro decatenation assay. Figure S8. Known Top1 inhibitor scaffolds: camptothecins (NSC94600), indenoisoquinolines (NSC314622), indolocarbazoles. Figure S9. Scaffold hopping candidates of camptothecin obtained using threshold values BA > 0.49, ROCS > 0.51, CFP ≤ 0.34. Figure S10. NSC24818 (podofilox). Figure S11. Scaffold hoppi [file 13321_2019_390_MOESM1_ESM.pdf]

Identifying new topoisomerase II poison scaffolds by combining publicly available toxicity data and 2D/3D-based virtual screening – Additional Information

Anna Lovrics[1], Veronika F.S. Pape[1,2], Dániel Szisz[3], Adrián Kalászi[3], Petra Heffeter[4], Csaba Magyar[1], Gergely Szakács[1,4*]

1 Institute of Enzymology, Research Centre for Natural Sciences, Hungarian Academy of Sciences, H-1117, Budapest, Hungary.

2 Current Address: Department of Physiology, Semmelweis University, Faculty of Medicine, H-1094 Budapest, Hungary

3 ChemAxon Ltd., Graphisoft park, Záhony u. 7, H-1031 Budapest, Hungary

4 Institute of Cancer Research and Comprehensive Cancer Center, Medical University of Vienna, Borschkegasse 8a, A-1090, Vienna, Austria.

* szakacs.gergely@ttk.mta.hu

Contents

1 Additional Text 3

2 Additional Figures 4

3 Additional Tables 10

1 Additional Text

Commands used for ROCS (OpenEye Scientific Software, Santa Fe, NM) simiarity calculations:

1) generate 3D conformers using OMEGA: omega2 -mpi_np <n> -in <input> -out <output> -prefix <P> -strictstereo false
n: number of processors n in MPI mode
input: the input sd file
output: the file containing the 3D conformers, used as input when running ROCS
strictstereo false: when stereocenters are not defined, random stereocenters are defined during the conformer generation
2) run ROCS to compare the structures: rocs -mpi_np <n> -query <qfile> -dbase <dfile> mcquery true -rankby TanimotoCombo -stats best -subrocs -scoreonly
n: number of processors n in MPI mode
mcquery: combine contiguous conformers in the query file into a multi-conformer query molecule
qfile: file containing the query molecule dfile: file containing the molecules to overlay against query
rankby ShapeTanimoto: rank molecular overlay by shape and color
stats best: the report includes stats for the best overlay(s) for every dbase molecule
subrocs: start the search at all heavy atoms of the larger molecule as well as the default inertial starts
scoreonly: perform scoring calculation only

Commands used for CFP (ChemAxon Ltd., Budapest, Hungary) similarity calculations:

screenmd <target.sdf> <query.sdf> -k CF -c <cfp.xml> -M Tanimoto -e 2 -o <output.txt>
target.sdf: sd file containing the target molecule(s)
query.sdf: sd file containing the query molecule(s)
-k CF: use the CFP descriptor
cfp.xml: the config file used, the cfp.xml available from jchem16.7/examples/config, where the Tanimoto threshold was set to 1.0, in order to report all similarity values
-M Tanimoto: use the metric Tanimoto as specified in the config file
-e 2: two decimal places after the decimal point
output.txt: the name of the output file

2 Additional Figures

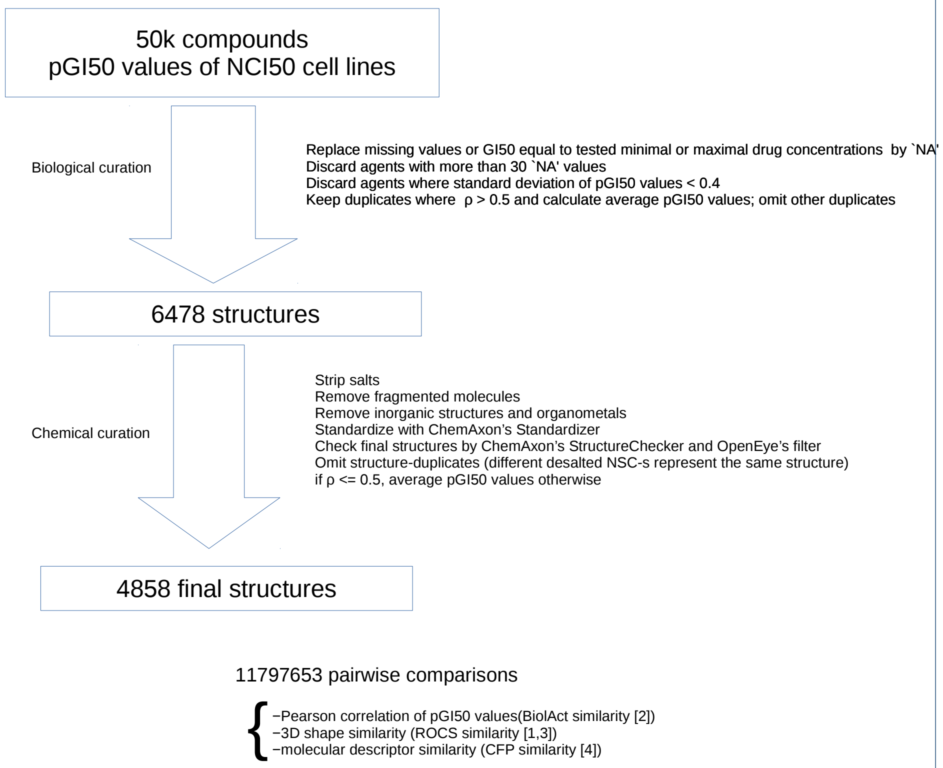

Figure S1: Flowchart depicting the selection and comparison of DTP molecules used in this study.

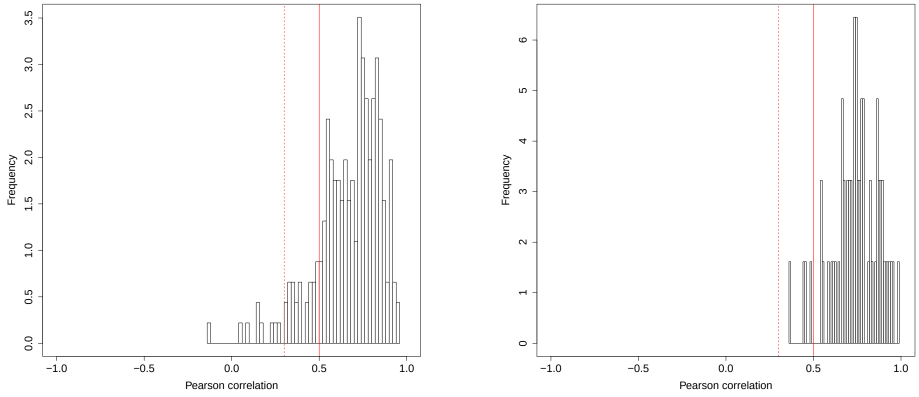

Figure S2: Histogram of pairwise Pearson correlation values among NSC duplicates (a) and desalted structure duplicates (b). Dashed vertical red line represents the Pearson correlation threshold used in this study to select scaffold-hopping analogues of mitoxantrone, while continuous red vertical line represents the cut-off for keeping duplicate structures.

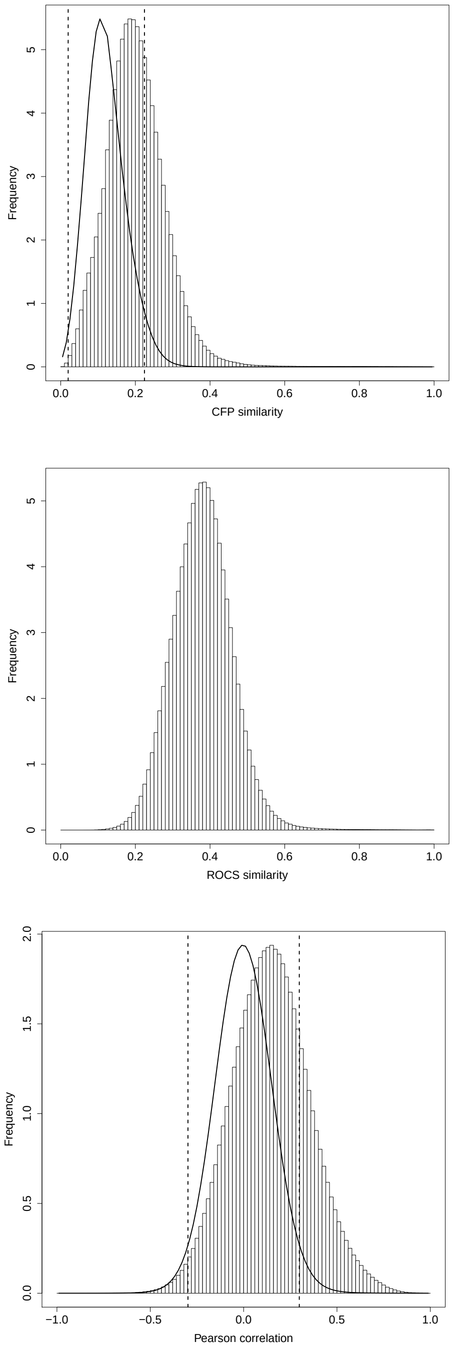

Figure S3: Distribution of the 11797653 pairwise similarity values supplemented with the bootstrapped distributions (continuous lines), where available. The vertical lines show the 95% confidence intervals of the bootstrapped distributions.

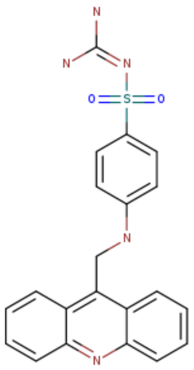

(a) NSC348894

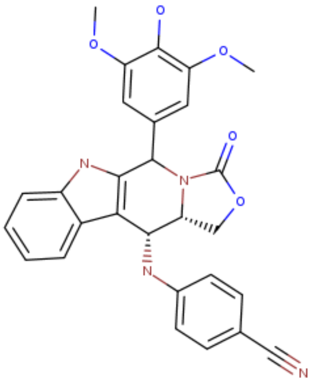

(b) NSC662305

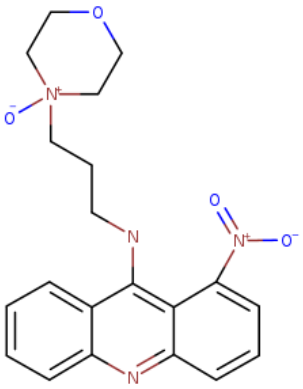

(c) NSC666735

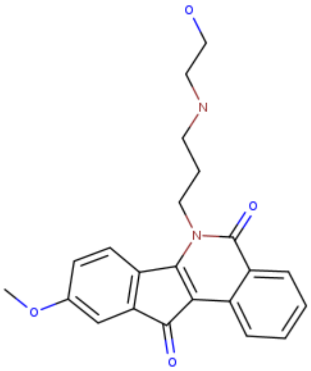

(d) NSC740650

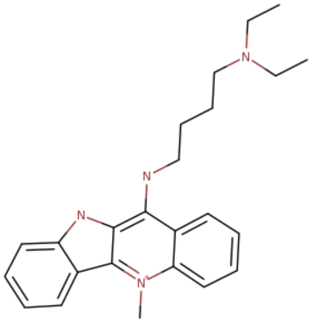

(e) NSC748393

Figure S4: Additional scaffold hopping candicates of either mitoxantrone, ametantrone, amsacrine or etoposide. Cf. Fig. 4 in main text.

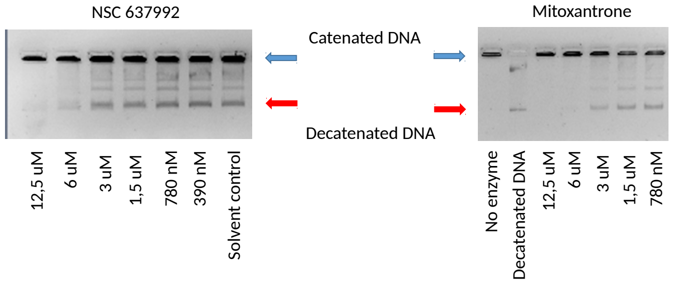

Figure S5: Example gel photos displaying dose-response Top2 poisoning of NSC637992 and mitoxantrone. Dose response curves were calculated based on the intensities corresponding to the decatenated DNA (red arrow).

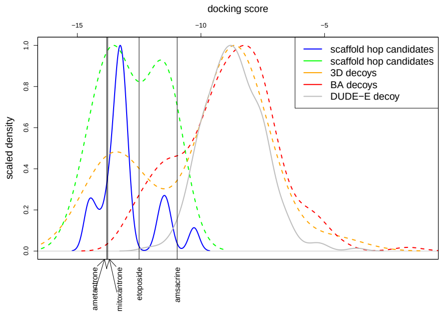

(a)

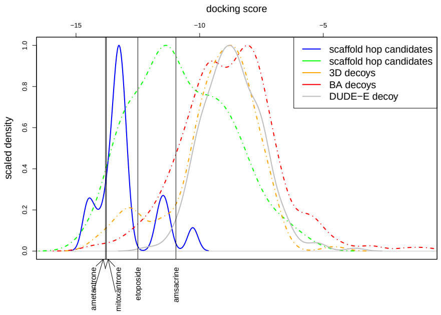

(b)

Figure S6: Scaled density of the docking scores calculated for candidate scaffold hopping analogues of mitoxantrone (blue), the '3D decoy' and the 'biological decoy' sets (orange and red, respectively) and the DUDE-E decoys (grey) when the similarity threshold values were chosen as the strictest (a) and most lenient (b).

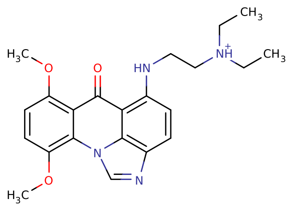

Figure S7: NSC660839, the '3D decoy' molecule tested in the *in vitro* decatenation assay.

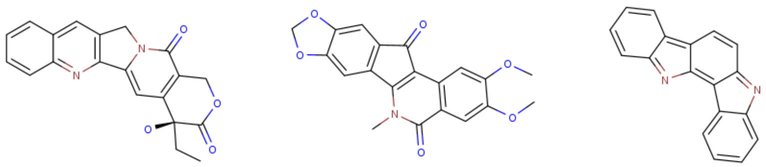

Figure S8: Known Top1 inhibitor scaffolds: camptothecins (NSC94600), indenoisoquinolines (NSC314622), indolocarbazoles.

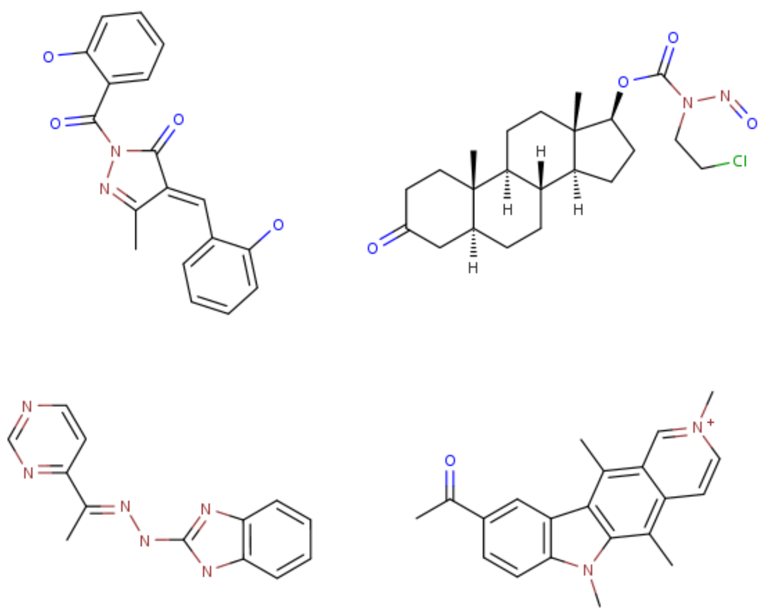

Figure S9: Scaffold hopping candidates of camptothecin obtained using threshold values BA $>$ 0.49, ROCS $>$ 0.51, CFP $<=$ 0.34.

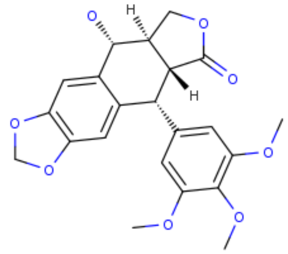

Figure S10: NSC24818 (podofilox).

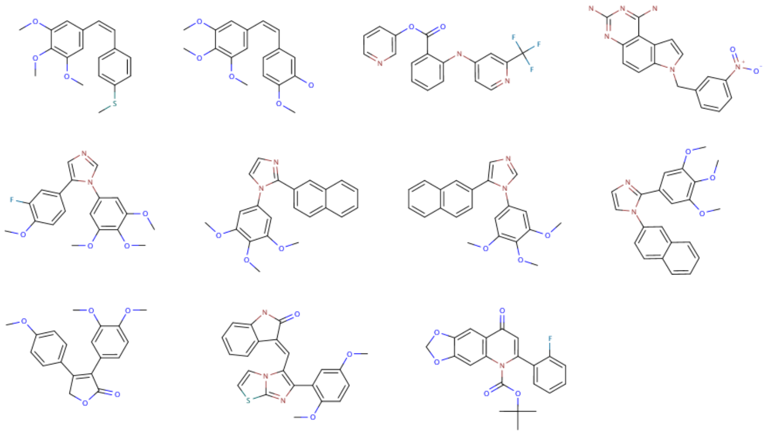

Figure S11: Scaffold hopping candidates of NSC24818 obtained using threshold values BA > 0.41, ROCS > 0.48, CFP <= 0.30.

3 Additional Tables

Table S1: Pearson correlation (BiolAct similarity) of the pIC50 values of annotated Top2 poisons to mitoxantrone.

name	NSC	BiolAct
ametantrone	196473	0.80
daunorubicin	83142	0.70
idarubicin	256439	0.72
N,N-dibenzyldaunorubicin	268242	0.43
epirubicin	256942	0.65
doxorubicin	123127	0.80
rubidazon	164011	0.83
valrubicin	246131	0.79
menogaril	269148	0.85
piroxantrone	349174	0.89
bisantrene	337766	0.61
amsacrine	249992	0.87
2-methylellipticinium	351710	0.44
2-N-Methyl-6-thiaellipticinium iodide	638066	0.59
etoposide	141540	0.66
teniposide	122819	0.86
dexrazoxane	169780	0.57

Table S2: Selected similarity thresholds to identify putative scaffold hopping analogues of mitoxantrone.

metric	threshold
BA	0.55
CFP	0.32
ROCS	0.51

Table S3: Docking scores and rankings (from 1173 compounds) of mitoxantrone and its scaffold hopping candidates.

chemotype	NSC	docking	rank
mitoxantrone	301739	-13.138	38
lucanthone derivatives	317003	-11.307	208
	317921	-11.474	187
	334352	-11.672	154
triazoloacridinones	645829	-13.412	24
	699148	-10.28	348
pyrimidoacridines	693117	-13.187	37
	693118	-14.476	2
	693119	-13.533	18
	693120	-14.573	1
	691849	-13.812	12
	691852	-14.172	7
sedoxantrone	635371	-13.322	32
imidazoacridinones	637991	-13.66	15
	637992	-13.254	35
	637994	-13.254	34
	645808	-13.326	31
	645809	-13.004	47
	645810	-13.269	33
5-substituted-9-aminoacridine 4 carboxamides	691240	-13.083	39
	693545	-12.952	52

Table S4: Maximum of biological, ROCS and 2D similarities compared to mitoxantrone, ametantrone, amsacrine or etoposide. Additionally, docking scores of these compounds.

NSC	BiolAct	ROCS	CFP	docking
348894	0.87	0.6	0.34	-11.23
662305	0.75	0.53	0.37	-12.72
666735	0.56	0.55	0.33	-9.47
740650	0.68	0.54	0.33	-13.00
748393	0.58	0.53	0.31	-12.12

Table S5: Similarities compared to published Top2 ligands and docking scores of 3D decoy agents.

NSC	lead	BiolAct	ROCS	CFP	docking
69600	amsacrine	0.07	0.54	0.32	-7.93
87034	amsacrine	0.10	0.57	0.28	-8.30
131734	amsacrine	0.43	0.59	0.32	-9.38
175636	amsacrine	0.43	0.54	0.18	-9.82
175639	amsacrine	0.34	0.54	0.24	-9.73
201873	amsacrine	-0.06	0.61	0.19	-8.82
208652	mitoxantrone	0.13	0.58	0.24	-6.67
225112	mitoxantrone	0.28	0.57	0.27	-7.93
280594	amsacrine	0.12	0.53	0.20	-8.56
348401	amsacrine	0.36	0.57	0.21	-7.12
348402	amsacrine	0.20	0.56	0.22	-7.38
601518	amsacrine	0.41	0.69	0.26	-9.34
612115	mitoxantrone	0.37	0.52	0.30	-7.10
625537	amsacrine	0.16	0.53	0.19	-9.59
627891	amsacrine	0.39	0.52	0.30	-8.09
637729	amsacrine	0.16	0.53	0.17	-8.53
637993	mitoxantrone	0.38	0.53	0.28	-12.47
645812	mitoxantrone	0.18	0.53	0.29	-12.72
645813	ametantrone	0.31	0.54	0.22	-12.97
645827	mitoxantrone	0.36	0.56	0.26	-12.74
645828	mitoxantrone	0.41	0.56	0.26	-12.30
645830	mitoxantrone	0.48	0.55	0.24	-12.91
647751	ametantrone	0.51	0.53	0.22	-12.02
648211	amsacrine	-0.03	0.52	0.25	-10.26
652183	amsacrine	0.31	0.54	0.18	-7.91
652884	amsacrine	0.26	0.53	0.33	-9.80
657348	ametantrone	0.20	0.59	0.24	-8.90
659998	mitoxantrone	0.14	0.54	0.31	-8.21
659999	mitoxantrone	0.14	0.52	0.32	-8.37
660638	mitoxantrone	0.23	0.53	0.33	-9.70
660839	mitoxantrone	0.13	0.61	0.28	-12.76
660840	mitoxantrone	0.19	0.61	0.27	-12.86
660841	mitoxantrone	0.39	0.55	0.29	-12.51
664000	amsacrine	0.06	0.52	0.18	-8.46
664878	amsacrine	0.39	0.53	0.21	-8.13
665378	amsacrine	0.19	0.59	0.23	-8.66
665492	amsacrine	0.28	0.53	0.22	-9.61
665700	amsacrine	0.01	0.52	0.21	-8.88
666735	amsacrine	0.29	0.55	0.33	-9.46
667645	amsacrine	-0.05	0.53	0.33	-10.62
668495	mitoxantrone	-0.10	0.61	0.27	-8.08
669381	amsacrine	0.43	0.56	0.22	-8.71
669888	amsacrine	0.16	0.57	0.22	-9.29
669943	amsacrine	0.10	0.52	0.22	-9.20
673350	ametantrone	0.37	0.56	0.32	-9.06
673805	amsacrine	0.11	0.53	0.33	-9.81
674220	mitoxantrone	0.25	0.55	0.25	-8.22
674256	amsacrine	0.31	0.56	0.20	-8.37
674257	amsacrine	0.35	0.57	0.20	-8.29
674258	amsacrine	0.44	0.60	0.25	-8.41
675370	amsacrine	0.10	0.55	0.22	-8.91
675985	mitoxantrone	0.39	0.53	0.24	-9.81
676514	amsacrine	0.12	0.56	0.30	-9.50
678866	amsacrine	0.29	0.53	0.19	-8.16
679813	mitoxantrone	0.33	0.56	0.12	-4.56
680781	amsacrine	0.25	0.53	0.15	-7.37
682011	amsacrine	0.08	0.54	0.28	-11.46
687308	mitoxantrone	0.29	0.56	0.21	-14.20
687311	amsacrine	0.24	0.77	0.26	-8.89
691850	mitoxantrone	0.51	0.57	0.24	-13.52
691851	mitoxantrone	0.27	0.56	0.25	-13.68
692309	amsacrine	0.03	0.52	0.22	-7.89
692646	amsacrine	0.28	0.65	0.27	-8.95
692738	ametantrone	0.24	0.52	0.30	-12.96
693246	amsacrine	0.34	0.52	0.25	-7.39
693255	mitoxantrone	-0.10	0.54	0.23	-6.78
693620	amsacrine	0.30	0.53	0.22	-7.59

Continued on next page

NSC	lead	BiolAct	ROCS	CFP	docking
695177	amsacrine	0.41	0.55	0.27	-7.28
696991	mitoxantrone	-0.00	0.53	0.31	-7.15
698959	mitoxantrone	0.38	0.54	0.24	-13.35
698960	mitoxantrone	0.39	0.56	0.23	-13.35
699147	ametantrone	0.23	0.53	0.22	-11.33
699149	ametantrone	0.44	0.53	0.21	-10.93
699152	mitoxantrone	0.40	0.54	0.26	-10.91
700415	amsacrine	0.17	0.53	0.19	-11.60
701023	amsacrine	0.26	0.52	0.22	-10.18
710546	mitoxantrone	0.28	0.54	0.20	-12.91
710547	mitoxantrone	0.03	0.53	0.23	-14.07
710548	mitoxantrone	0.46	0.53	0.23	-14.02
710549	ametantrone	0.15	0.53	0.21	-13.37
711725	amsacrine	0.27	0.56	0.30	-8.44
719923	amsacrine	0.33	0.58	0.22	-8.06
720558	amsacrine	-0.00	0.70	0.34	-10.69
720563	amsacrine	0.02	0.53	0.30	-8.55
720570	amsacrine	0.16	0.77	0.33	-10.19
720688	mitoxantrone	0.11	0.53	0.24	-12.74
723394	mitoxantrone	0.24	0.53	0.28	-6.96
723423	ametantrone	0.25	0.70	0.17	-9.06
724686	amsacrine	0.30	0.70	0.33	-9.55
724693	amsacrine	0.04	0.64	0.28	-9.92
724699	amsacrine	0.30	0.58	0.33	-9.11
726438	ametantrone	0.10	0.53	0.31	-11.17
727450	amsacrine	0.05	0.55	0.33	-10.20
727885	amsacrine	0.21	0.54	0.18	-9.01
731164	mitoxantrone	0.23	0.53	0.24	-8.42
734631	amsacrine	0.26	0.53	0.29	-9.11
736793	amsacrine	0.30	0.52	0.18	-9.89
740650	ametantrone	0.51	0.54	0.28	-13.00
744231	ametantrone	0.36	0.55	0.22	-11.51
745450	amsacrine	0.22	0.55	0.24	-8.58
745455	amsacrine	0.27	0.54	0.24	-8.66
745750	mitoxantrone	-0.18	0.53	0.26	-9.00
748392	amsacrine	0.20	0.54	0.31	-12.65
748393	amsacrine	0.25	0.53	0.31	-12.12
748533	amsacrine	0.18	0.58	0.22	-8.64
748870	amsacrine	0.24	0.52	0.23	-10.24
749804	amsacrine	0.16	0.55	0.22	-8.70
751830	amsacrine	-0.03	0.56	0.28	-8.62

Table S6: Similarities compared to published Top2 ligands and docking scores of biological activity decoy agents.

NSC	lead	BiolAct	ROCS	CFP	docking
762	mitoxantrone	0.60	0.17	0.05	-4.67
3424	mitoxantrone	0.59	0.42	0.27	-10.14
4623	etoposide	0.61	0.27	0.15	-6.89
6171	ametantrone	0.55	0.24	0.06	-5.42
6396	mitoxantrone	0.72	0.19	0.05	-5.23
9706	mitoxantrone	0.73	0.36	0.08	-6.15
12150	ametantrone	0.55	0.34	0.26	-10.37
12455	ametantrone	0.53	0.30	0.22	-4.38
19781	etoposide	0.61	0.33	0.16	-6.71
22709	etoposide	0.55	0.17	0.10	-5.97
22984	etoposide	0.59	0.21	0.16	-5.97
26821	etoposide	0.60	0.28	0.11	-7.81
26980	mitoxantrone	0.74	0.40	0.20	-9.46
29228	etoposide	0.57	0.22	0.02	-2.96
30706	etoposide	0.61	0.21	0.11	-7.12
31717	amsacrine	0.58	0.31	0.08	-7.78
32065	mitoxantrone	0.54	0.14	0.04	-5.38
34462	mitoxantrone	0.72	0.30	0.14	-7.57
35915	mitoxantrone	0.69	0.25	0.11	-8.60
48006	amsacrine	0.52	0.40	0.16	-8.21

Continued on next page

NSC	lead	BiolAct	ROCS	CFP	docking
48034	mitoxantrone	0.70	0.32	0.24	-8.07
48956	mitoxantrone	0.60	0.29	0.25	-8.05
49520	etoposide	0.52	0.24	0.14	-8.13
50273	amsacrine	0.52	0.41	0.22	-7.92
56410	mitoxantrone	0.69	0.39	0.20	-10.48
57199	mitoxantrone	0.66	0.34	0.31	-8.52
58514	mitoxantrone	0.53	0.10	0.28	-8.59
62709	mitoxantrone	0.56	0.34	0.24	-6.72
63878	mitoxantrone	0.62	0.31	0.17	-8.48
64703	mitoxantrone	0.64	0.34	0.24	-8.01
72151	amsacrine	0.61	0.31	0.23	-7.98
73735	etoposide	0.62	0.32	0.25	-7.35
75520	mitoxantrone	0.54	0.39	0.19	-8.47
76919	mitoxantrone	0.60	0.34	0.27	-7.46
79037	etoposide	0.59	0.24	0.07	-5.56
80103	mitoxantrone	0.61	0.28	0.11	-8.60
81767	amsacrine	0.67	0.39	0.21	-8.38
82587	mitoxantrone	0.60	0.31	0.29	-9.52
83343	amsacrine	0.56	0.39	0.21	-7.88
85230	mitoxantrone	0.72	0.35	0.25	-8.92
86371	etoposide	0.59	0.31	0.22	-10.25
90810	etoposide	0.63	0.30	0.14	-7.67
92497	etoposide	0.62	0.22	0.13	-6.54
92498	etoposide	0.57	0.21	0.14	-6.97
92499	etoposide	0.57	0.20	0.14	-6.66
94600	mitoxantrone	0.63	0.37	0.27	-9.63
95382	mitoxantrone	0.73	0.35	0.26	-8.78
95466	mitoxantrone	0.60	0.31	0.12	-6.68
98572	amsacrine	0.53	0.43	0.18	-8.35
99445	mitoxantrone	0.53	0.36	0.18	-8.95
103704	amsacrine	0.54	0.40	0.15	-8.03
105132	mitoxantrone	0.69	0.37	0.27	-9.42
107124	mitoxantrone	0.68	0.42	0.27	-10.70
107392	etoposide	0.53	0.23	0.13	-7.96
111533	mitoxantrone	0.74	0.37	0.27	-9.34
112758	ametantrone	0.70	0.40	0.28	-10.33
112965	amsacrine	0.67	0.43	0.17	-7.36
114346	etoposide	0.56	0.33	0.30	-8.51
122870	amsacrine	0.57	0.35	0.24	-7.95
123115	mitoxantrone	0.66	0.39	0.20	-9.32
124147	ametantrone	0.58	0.36	0.21	-7.76
126849	amsacrine	0.54	0.36	0.13	-7.87
127763	etoposide	0.62	0.33	0.24	-9.14
128687	etoposide	0.65	0.33	0.26	-7.58
129943	amsacrine	0.58	0.38	0.06	-8.43
131547	ametantrone	0.56	0.36	0.23	-10.88
132313	mitoxantrone	0.70	0.31	0.05	-4.38
134459	etoposide	0.59	0.25	0.12	-6.90
134679	mitoxantrone	0.52	0.41	0.17	-8.70
134723	mitoxantrone	0.62	0.40	0.20	-9.10
134725	mitoxantrone	0.62	0.40	0.20	-9.19
134727	mitoxantrone	0.65	0.46	0.20	-9.51
135758	mitoxantrone	0.65	0.32	0.11	-9.45
135962	ametantrone	0.55	0.33	0.15	-7.55
138429	etoposide	0.55	0.39	0.31	-7.38
140701	mitoxantrone	0.71	0.34	0.18	-11.32
141537	etoposide	0.53	0.30	0.27	-6.98
143095	amsacrine	0.54	0.41	0.18	-6.97
143491	ametantrone	0.67	0.40	0.28	-11.57
145668	amsacrine	0.72	0.37	0.16	-9.29
146268	amsacrine	0.53	0.40	0.14	-8.66
146396	mitoxantrone	0.53	0.36	0.33	-8.88
146397	ametantrone	0.56	0.35	0.18	-10.19
149584	ametantrone	0.70	0.35	0.27	-11.08
150862	mitoxantrone	0.52	0.16	0.24	-11.87
152731	ametantrone	0.54	0.43	0.19	-10.11
154318	mitoxantrone	0.70	0.41	0.22	-10.71
155693	ametantrone	0.53	0.39	0.19	-11.06
155694	ametantrone	0.59	0.42	0.19	-11.62
156305	mitoxantrone	0.65	0.40	0.18	-10.58

Continued on next page

NSC	lead	BiolAct	ROCS	CFP	docking
157995	ametantrone	0.53	0.37	0.16	-10.20
162062	etoposide	0.53	0.26	0.12	-6.48
166199	mitoxantrone	0.66	0.31	0.10	-6.74
166224	amsacrine	0.56	0.34	0.21	-7.36
166237	etoposide	0.69	0.32	0.23	-8.08
166641	mitoxantrone	0.54	0.33	0.18	-9.54
167780	etoposide	0.69	0.29	0.28	-7.89
169534	ametantrone	0.68	0.40	0.28	-10.37
176323	amsacrine	0.70	0.40	0.26	-8.73
176878	ametantrone	0.54	0.27	0.20	-7.18
177365	ametantrone	0.53	0.34	0.20	-10.98
178248	mitoxantrone	0.57	0.32	0.14	-7.46
180510	ametantrone	0.60	0.40	0.29	-11.83
182986	mitoxantrone	0.68	0.38	0.13	-7.13
184692	amsacrine	0.54	0.35	0.23	-7.99
196473	etoposide	0.64	0.39	0.23	-13.45
201290	etoposide	0.58	0.30	0.25	-8.77
207895	mitoxantrone	0.55	0.40	0.26	-9.36
221265	ametantrone	0.68	0.34	0.28	-12.38
234714	amsacrine	0.69	0.44	0.07	-6.66
237538	mitoxantrone	0.53	0.30	0.17	-7.81
239336	etoposide	0.71	0.26	0.25	-6.98
243928	mitoxantrone	0.80	0.37	0.21	-11.01
243929	mitoxantrone	0.69	0.36	0.21	-10.82
243930	mitoxantrone	0.72	0.35	0.20	-9.46
249910	mitoxantrone	0.58	0.37	0.26	-8.71
249911	mitoxantrone	0.67	0.38	0.26	-9.72
249992	ametantrone	0.64	0.39	0.19	-10.94
250684	mitoxantrone	0.55	0.28	0.19	-9.35
250686	etoposide	0.60	0.29	0.25	-7.85
256438	amsacrine	0.69	0.37	0.18	-10.74
259968	ametantrone	0.53	0.26	0.25	-6.53
259969	ametantrone	0.59	0.26	0.24	-5.81
260610	ametantrone	0.56	0.29	0.23	-11.24
266068	mitoxantrone	0.52	0.31	0.26	-9.24
267707	etoposide	0.57	0.30	0.29	-6.83
268965	amsacrine	0.65	0.38	0.19	-7.78
269719	mitoxantrone	0.58	0.35	0.16	-8.46
269720	amsacrine	0.55	0.42	0.12	-6.79
269721	mitoxantrone	0.58	0.35	0.15	-7.75
275656	mitoxantrone	0.70	0.35	0.13	-6.93
280074	etoposide	0.56	0.27	0.28	-8.37
281272	mitoxantrone	0.55	0.33	0.16	-8.37
281612	amsacrine	0.62	0.41	0.19	-8.32
281613	mitoxantrone	0.57	0.27	0.27	-7.69
281617	mitoxantrone	0.54	0.27	0.28	-9.05
281817	mitoxantrone	0.53	0.27	0.27	-9.44
281818	mitoxantrone	0.53	0.26	0.29	-7.19
284682	amsacrine	0.56	0.35	0.19	-9.78
286628	ametantrone	0.54	0.37	0.27	-11.34
292463	etoposide	0.57	0.29	0.29	-5.93
294980	mitoxantrone	0.67	0.28	0.21	-6.99
295499	mitoxantrone	0.76	0.39	0.26	-9.41
295500	mitoxantrone	0.83	0.38	0.27	-9.27
295501	mitoxantrone	0.53	0.38	0.27	-10.03
296934	mitoxantrone	0.62	0.40	0.11	-5.44
300288	mitoxantrone	0.76	0.45	0.28	-11.14
301477	ametantrone	0.62	0.39	0.27	-11.09
301480	amsacrine	0.52	0.38	0.18	-8.67
301739	etoposide	0.75	0.38	0.31	-13.14
302325	amsacrine	0.53	0.40	0.17	-8.49
302991	amsacrine	0.66	0.39	0.25	-9.63
303812	mitoxantrone	0.61	0.41	0.11	-7.98
307990	ametantrone	0.56	0.37	0.26	-10.84
314622	mitoxantrone	0.55	0.39	0.33	-9.64
319726	amsacrine	0.53	0.41	0.16	-7.63
324646	ametantrone	0.64	0.29	0.12	-8.67
328410	mitoxantrone	0.78	0.37	0.26	-8.88
331269	mitoxantrone	0.61	0.40	0.14	-5.88
332488	amsacrine	0.67	0.38	0.13	-5.36

Continued on next page

NSC	lead	BiolAct	ROCS	CFP	docking
333054	amsacrine	0.77	0.37	0.19	-10.52
335766	etoposide	0.57	0.30	0.27	-7.76
339705	mitoxantrone	0.62	0.41	0.30	-10.40
342443	ametantrone	0.55	0.26	0.15	-11.05
344007	mitoxantrone	0.71	0.28	0.08	-6.84
344505	amsacrine	0.63	0.43	0.19	-9.25
348115	amsacrine	0.52	0.40	0.23	-10.09
348894	mitoxantrone	0.85	0.40	0.23	-11.23
350378	etoposide	0.54	0.36	0.25	-10.00
351520	etoposide	0.54	0.33	0.24	-9.22
353076	ametantrone	0.56	0.43	0.20	-12.63
355457	mitoxantrone	0.53	0.37	0.34	-11.51
357885	etoposide	0.70	0.41	0.30	-14.19
360702	mitoxantrone	0.63	0.40	0.17	-7.97
364830	mitoxantrone	0.72	0.42	0.26	-9.39
365360	ametantrone	0.69	0.30	0.25	-11.64
367428	amsacrine	0.66	0.34	0.09	-5.75
369395	amsacrine	0.60	0.38	0.24	-10.73
374028	mitoxantrone	0.69	0.40	0.27	-11.60
375575	amsacrine	0.59	0.40	0.16	-8.07
376254	mitoxantrone	0.58	0.36	0.30	-8.15
376739	ametantrone	0.65	0.40	0.27	-9.25
378901	ametantrone	0.61	0.39	0.27	-10.08
380212	amsacrine	0.53	0.40	0.18	-8.85
382001	etoposide	0.52	0.24	0.18	-7.73
382035	amsacrine	0.56	0.43	0.19	-8.43
400492	etoposide	0.58	0.26	0.16	-7.86
407335	mitoxantrone	0.56	0.33	0.17	-8.41
529180	amsacrine	0.60	0.40	0.17	-8.21
600167	mitoxantrone	0.59	0.28	0.18	-7.28
603071	amsacrine	0.60	0.38	0.25	-10.20
606172	mitoxantrone	0.66	0.40	0.27	-10.17
606173	mitoxantrone	0.77	0.43	0.26	-9.34
606174	amsacrine	0.55	0.37	0.25	-10.16
606497	mitoxantrone	0.69	0.42	0.26	-9.32
606498	mitoxantrone	0.62	0.41	0.26	-9.55
606499	mitoxantrone	0.82	0.42	0.26	-10.21
606985	mitoxantrone	0.58	0.39	0.26	-10.23
606986	amsacrine	0.67	0.37	0.25	-9.87
607347	mitoxantrone	0.57	0.33	0.08	-7.52
609699	mitoxantrone	0.67	0.38	0.28	-9.76
610456	mitoxantrone	0.56	0.38	0.26	-7.85
610457	mitoxantrone	0.57	0.40	0.26	-10.47
610458	mitoxantrone	0.79	0.34	0.26	-6.72
610459	mitoxantrone	0.67	0.40	0.26	-9.31
612828	amsacrine	0.58	0.43	0.14	-8.14
613327	mitoxantrone	0.54	0.34	0.19	-7.95
614049	etoposide	0.56	0.36	0.21	-11.78
616348	mitoxantrone	0.72	0.31	0.27	-10.52
617208	etoposide	0.57	0.22	0.20	-8.09
617806	etoposide	0.62	0.23	0.05	-7.21
617953	ametantrone	0.52	0.43	0.25	-10.00
618939	mitoxantrone	0.67	0.42	0.26	-9.12
619003	amsacrine	0.84	0.35	0.18	-9.24
620480	mitoxantrone	0.61	0.29	0.25	-8.71
621199	etoposide	0.53	0.41	0.30	-10.14
623128	amsacrine	0.68	0.36	0.18	-9.84
623872	etoposide	0.65	0.26	0.20	-7.49
623905	etoposide	0.54	0.38	0.28	-8.11
624158	etoposide	0.58	0.33	0.12	-7.14
624414	etoposide	0.57	0.36	0.28	-9.66
624431	etoposide	0.57	0.41	0.32	-9.10
624432	etoposide	0.61	0.37	0.28	-8.25
624434	etoposide	0.52	0.27	0.28	-8.62
624851	etoposide	0.62	0.17	0.05	-5.45
625156	etoposide	0.53	0.28	0.22	-3.21
625174	etoposide	0.53	0.22	0.10	-6.86
626540	etoposide	0.53	0.37	0.21	-11.54
628497	mitoxantrone	0.63	0.43	0.28	-9.96
628498	mitoxantrone	0.53	0.44	0.26	-11.87

Continued on next page

NSC	lead	BiolAct	ROCS	CFP	docking
628662	amsacrine	0.87	0.37	0.22	-11.71
628663	ametantrone	0.60	0.39	0.28	-12.42
628664	ametantrone	0.57	0.37	0.29	-12.38
628665	mitoxantrone	0.70	0.38	0.34	-11.87
628666	mitoxantrone	0.70	0.37	0.33	-11.80
628667	amsacrine	0.83	0.36	0.22	-11.69
628668	ametantrone	0.55	0.38	0.27	-12.48
628669	amsacrine	0.90	0.38	0.21	-12.20
628670	ametantrone	0.53	0.38	0.29	-12.21
628671	amsacrine	0.83	0.39	0.21	-12.21
628672	ametantrone	0.60	0.38	0.29	-12.15
628673	amsacrine	0.73	0.36	0.22	-10.04
628674	amsacrine	0.83	0.37	0.22	-11.66
628675	amsacrine	0.82	0.36	0.21	-12.49
628676	ametantrone	0.61	0.38	0.28	-11.57
628677	amsacrine	0.79	0.37	0.22	-11.71
628678	amsacrine	0.87	0.36	0.22	-12.80
628679	mitoxantrone	0.76	0.37	0.34	-11.57
628680	amsacrine	0.86	0.38	0.21	-11.60
628681	amsacrine	0.53	0.38	0.20	-11.20
628908	etoposide	0.55	0.26	0.19	-8.25
628918	etoposide	0.59	0.32	0.23	-10.70
629266	mitoxantrone	0.53	0.39	0.26	-9.44
629874	etoposide	0.54	0.34	0.21	-7.60
629971	mitoxantrone	0.65	0.37	0.26	-10.20
630604	etoposide	0.55	0.33	0.21	-7.53
630699	mitoxantrone	0.52	0.46	0.27	-12.68
630700	mitoxantrone	0.66	0.42	0.26	-12.01
630701	ametantrone	0.54	0.38	0.24	-12.55
630702	mitoxantrone	0.75	0.38	0.26	-11.79
631197	mitoxantrone	0.62	0.43	0.26	-11.72
631199	ametantrone	0.56	0.44	0.25	-11.18
631939	amsacrine	0.61	0.32	0.07	-8.38
633403	etoposide	0.54	0.33	0.27	-11.24
634458	etoposide	0.62	0.25	0.06	-0.81
634724	amsacrine	0.77	0.37	0.25	-10.16
635405	etoposide	0.56	0.33	0.30	-7.60
637126	amsacrine	0.54	0.41	0.31	-10.37
637140	mitoxantrone	0.55	0.42	0.27	-9.12
637611	ametantrone	0.56	0.29	0.30	-8.02
637651	ametantrone	0.54	0.38	0.21	-9.60
638404	mitoxantrone	0.52	0.36	0.20	-7.65
638441	mitoxantrone	0.52	0.36	0.17	-9.08
638856	mitoxantrone	0.57	0.29	0.19	-8.93
639174	mitoxantrone	0.60	0.37	0.27	-9.81
639176	amsacrine	0.70	0.38	0.26	-9.46
639519	etoposide	0.53	0.29	0.18	-9.73
639659	ametantrone	0.65	0.38	0.27	-9.69
639831	mitoxantrone	0.58	0.34	0.20	-10.25
639832	mitoxantrone	0.61	0.32	0.20	-10.22
640085	ametantrone	0.61	0.30	0.21	-7.63
641454	amsacrine	0.54	0.39	0.23	-8.60
641533	etoposide	0.54	0.19	0.07	-6.05
642276	amsacrine	0.59	0.38	0.22	-11.43
642277	ametantrone	0.59	0.44	0.28	-11.28
642278	mitoxantrone	0.84	0.39	0.34	-11.29
642279	mitoxantrone	0.83	0.40	0.32	-12.74
642280	mitoxantrone	0.74	0.41	0.34	-11.61
642281	mitoxantrone	0.76	0.41	0.31	-11.37
642282	mitoxantrone	0.75	0.40	0.31	-13.08
642283	mitoxantrone	0.56	0.40	0.31	-12.92
642284	mitoxantrone	0.76	0.42	0.31	-12.77
642285	mitoxantrone	0.76	0.38	0.34	-10.96
642286	mitoxantrone	0.73	0.39	0.33	-11.09
642287	mitoxantrone	0.74	0.42	0.31	-13.43
642288	mitoxantrone	0.53	0.40	0.33	-10.71
642289	mitoxantrone	0.77	0.40	0.31	-11.32
642290	mitoxantrone	0.73	0.38	0.34	-11.21
642291	mitoxantrone	0.73	0.40	0.32	-9.42
642292	mitoxantrone	0.67	0.38	0.32	-11.54

Continued on next page

NSC	lead	BiolAct	ROCS	CFP	docking
642293	mitoxantrone	0.69	0.40	0.32	-11.23
642318	amsacrine	0.53	0.40	0.21	-8.44
642321	etoposide	0.58	0.38	0.25	-7.98
642322	mitoxantrone	0.80	0.45	0.30	-11.64
642323	mitoxantrone	0.79	0.42	0.30	-11.96
642324	mitoxantrone	0.80	0.41	0.29	-11.24
642328	ametantrone	0.57	0.38	0.28	-10.79
642329	ametantrone	0.56	0.36	0.28	-10.54
642911	mitoxantrone	0.57	0.31	0.16	-7.33
642915	mitoxantrone	0.75	0.31	0.16	-8.69
643001	etoposide	0.56	0.31	0.22	-5.92
643006	mitoxantrone	0.65	0.33	0.16	-7.50
643833	mitoxantrone	0.55	0.39	0.26	-9.52
644945	mitoxantrone	0.79	0.46	0.33	-11.50
644946	mitoxantrone	0.81	0.44	0.33	-12.49
644947	ametantrone	0.60	0.43	0.28	-11.67
644960	mitoxantrone	0.67	0.32	0.19	-9.10
644961	mitoxantrone	0.66	0.31	0.18	-8.95
644967	amsacrine	0.57	0.36	0.11	-7.93
645008	mitoxantrone	0.57	0.44	0.24	-11.91
645017	etoposide	0.73	0.31	0.30	-13.23
645018	etoposide	0.79	0.37	0.31	-13.57
645737	mitoxantrone	0.79	0.42	0.33	-12.30
646714	amsacrine	0.76	0.34	0.08	-7.68
648114	mitoxantrone	0.74	0.39	0.21	-10.29
648147	etoposide	0.54	0.30	0.21	-10.20
648316	mitoxantrone	0.58	0.40	0.19	-8.26
649677	mitoxantrone	0.80	0.39	0.22	-12.82
650259	ametantrone	0.53	0.42	0.18	-10.57
650931	ametantrone	0.60	0.39	0.27	-10.65
651777	amsacrine	0.54	0.49	0.19	-9.01
651850	mitoxantrone	0.73	0.43	0.32	-12.07
651855	mitoxantrone	0.67	0.40	0.32	-12.77
651857	mitoxantrone	0.71	0.42	0.33	-12.88
651861	mitoxantrone	0.75	0.42	0.32	-12.27
653438	mitoxantrone	0.58	0.47	0.25	-12.67
653842	amsacrine	0.54	0.44	0.17	-8.74
653860	mitoxantrone	0.78	0.47	0.32	-13.37
653862	amsacrine	0.55	0.12	0.22	-9.68
653863	mitoxantrone	0.67	0.43	0.32	-12.57
653864	ametantrone	0.54	0.45	0.25	-12.57
654509	amsacrine	0.77	0.36	0.19	-10.13
654830	amsacrine	0.56	0.38	0.20	-9.24
656903	etoposide	0.52	0.39	0.33	-7.68
656904	etoposide	0.53	0.38	0.32	-7.38
657339	etoposide	0.58	0.31	0.22	-7.83
657446	etoposide	0.53	0.37	0.27	-9.88
658229	etoposide	0.55	0.27	0.18	-8.06
659687	mitoxantrone	0.79	0.44	0.30	-11.68
659948	amsacrine	0.83	0.36	0.18	-11.12
659949	amsacrine	0.73	0.36	0.18	-9.98
659950	ametantrone	0.58	0.40	0.25	-9.48
660027	ametantrone	0.60	0.43	0.29	-11.03
660028	ametantrone	0.61	0.41	0.30	-11.07
660029	ametantrone	0.57	0.40	0.28	-11.40
661226	etoposide	0.53	0.34	0.28	-9.66
662305	mitoxantrone	0.73	0.40	0.29	-12.72
663249	etoposide	0.63	0.25	0.11	-7.60
663291	etoposide	0.62	0.32	0.30	-8.91
663790	amsacrine	0.59	0.42	0.15	-8.02
663791	amsacrine	0.58	0.43	0.15	-7.64
665350	etoposide	0.62	0.35	0.30	-9.71
665534	mitoxantrone	0.61	0.13	0.29	-1.61
665934	mitoxantrone	0.80	0.44	0.32	-12.80
665935	mitoxantrone	0.77	0.47	0.33	-13.55
666871	amsacrine	0.53	0.39	0.16	-8.08
667047	amsacrine	0.59	0.40	0.20	-8.83
667070	etoposide	0.57	0.25	0.17	-6.53
667641	amsacrine	0.56	0.28	0.21	-8.96
667870	amsacrine	0.54	0.34	0.15	-7.16

Continued on next page

NSC	lead	BiolAct	ROCS	CFP	docking
668281	mitoxantrone	0.61	0.32	0.17	-7.93
668352	etoposide	0.52	0.27	0.16	-8.43
668370	etoposide	0.55	0.19	0.12	-6.51
668380	mitoxantrone	0.77	0.40	0.30	-12.81
668404	mitoxantrone	0.55	0.27	0.23	-7.92
668523	mitoxantrone	0.64	0.19	0.05	-5.47
668605	etoposide	0.57	0.30	0.21	-11.49
668834	etoposide	0.54	0.32	0.30	-7.01
668918	etoposide	0.59	0.30	0.27	-5.90
669727	mitoxantrone	0.58	0.42	0.18	-8.12
669728	mitoxantrone	0.54	0.17	0.18	-8.46
670013	mitoxantrone	0.67	0.30	0.31	-9.62
670430	etoposide	0.54	0.37	0.22	-8.09
670653	amsacrine	0.61	0.40	0.16	-8.39
670654	amsacrine	0.53	0.34	0.17	-9.60
670656	amsacrine	0.76	0.39	0.18	-7.90
670807	etoposide	0.53	0.24	0.08	-6.20
671002	mitoxantrone	0.57	0.34	0.14	-7.30
671014	etoposide	0.59	0.32	0.22	-9.26
671331	etoposide	0.57	0.26	0.25	-7.80
672974	etoposide	0.57	0.30	0.24	-8.13
672975	mitoxantrone	0.57	0.30	0.19	-8.12
673150	etoposide	0.60	0.27	0.19	-4.53
673347	etoposide	0.59	0.35	0.15	-5.93
673348	etoposide	0.54	0.34	0.24	-8.95
673804	ametantrone	0.56	0.43	0.25	-11.35
673831	amsacrine	0.58	0.41	0.16	-6.86
673833	amsacrine	0.66	0.36	0.15	-7.17
673841	etoposide	0.55	0.26	0.19	-9.25
674182	amsacrine	0.65	0.39	0.17	-7.69
675249	mitoxantrone	0.64	0.32	0.28	-11.13
675250	mitoxantrone	0.65	0.30	0.28	-11.74
675251	mitoxantrone	0.53	0.32	0.30	-10.73
675255	mitoxantrone	0.65	0.36	0.31	-9.78
675256	mitoxantrone	0.53	0.28	0.25	-7.99
675574	mitoxantrone	0.69	0.49	0.19	-10.67
676316	etoposide	0.54	0.37	0.22	-8.25
678532	etoposide	0.58	0.31	0.24	-7.65
678880	etoposide	0.53	0.35	0.21	-7.92
678882	etoposide	0.53	0.40	0.22	-8.93
678914	mitoxantrone	0.59	0.49	0.25	-10.89
679108	etoposide	0.53	0.36	0.17	-8.51
679265	etoposide	0.55	0.36	0.23	-10.27
680417	mitoxantrone	0.57	0.17	0.20	-10.01
680420	mitoxantrone	0.55	0.30	0.20	-10.20
680715	etoposide	0.53	0.18	0.13	-7.72
680717	etoposide	0.54	0.31	0.23	-8.06
681152	etoposide	0.57	0.33	0.25	-8.71
681324	etoposide	0.54	0.27	0.20	-6.80
681326	etoposide	0.52	0.26	0.29	-9.34
681462	amsacrine	0.57	0.37	0.21	-9.68
681632	mitoxantrone	0.66	0.36	0.27	-10.73
681633	mitoxantrone	0.67	0.41	0.27	-11.05
681634	amsacrine	0.65	0.39	0.23	-8.30
681635	amsacrine	0.69	0.39	0.24	-10.66
681636	mitoxantrone	0.67	0.39	0.27	-10.49
681637	mitoxantrone	0.66	0.39	0.27	-10.68
681638	mitoxantrone	0.66	0.39	0.26	-10.73
681639	mitoxantrone	0.69	0.39	0.26	-10.76
681640	mitoxantrone	0.61	0.36	0.26	-10.76
681641	mitoxantrone	0.77	0.43	0.27	-9.95
681642	mitoxantrone	0.76	0.39	0.27	-10.60
681643	mitoxantrone	0.70	0.37	0.26	-10.20
681644	mitoxantrone	0.74	0.44	0.27	-10.10
681645	mitoxantrone	0.75	0.38	0.27	-9.59
681646	mitoxantrone	0.64	0.39	0.27	-10.13
682298	mitoxantrone	0.69	0.36	0.26	-10.12
682306	amsacrine	0.56	0.42	0.21	-8.78
683140	amsacrine	0.79	0.14	0.22	-13.39
683247	amsacrine	0.56	0.47	0.33	-13.36

Continued on next page

NSC	lead	BiolAct	ROCS	CFP	docking
683252	mitoxantrone	0.62	0.41	0.26	-6.47
683414	amsacrine	0.86	0.37	0.21	-9.86
683415	mitoxantrone	0.69	0.37	0.32	-9.86
683416	amsacrine	0.63	0.11	0.21	-9.07
683556	mitoxantrone	0.56	0.08	0.27	-7.78
683558	mitoxantrone	0.67	0.21	0.27	-10.66
684667	mitoxantrone	0.68	0.48	0.24	-11.17
684902	mitoxantrone	0.56	0.09	0.26	-5.42
684906	mitoxantrone	0.56	0.10	0.24	-5.57
684907	mitoxantrone	0.59	0.09	0.25	-6.88
684908	mitoxantrone	0.57	0.09	0.26	-6.81
684913	amsacrine	0.53	0.38	0.18	-8.64
685803	amsacrine	0.55	0.47	0.23	-7.57
687305	amsacrine	0.59	0.41	0.27	-8.49
687353	mitoxantrone	0.57	0.47	0.21	-11.27
688021	mitoxantrone	0.56	0.31	0.15	-6.89
688022	mitoxantrone	0.56	0.32	0.20	-6.30
688274	etoposide	0.54	0.36	0.34	-5.15
690266	etoposide	0.58	0.35	0.30	-9.53
690269	etoposide	0.62	0.36	0.31	-10.15
690760	etoposide	0.57	0.35	0.30	-8.73
690983	etoposide	0.57	0.35	0.30	-7.00
691244	amsacrine	0.56	0.43	0.26	-8.77
691247	mitoxantrone	0.65	0.41	0.25	-11.74
691249	mitoxantrone	0.64	0.42	0.26	-12.00
691251	amsacrine	0.61	0.40	0.29	-12.20
691277	etoposide	0.52	0.34	0.23	-10.09
691656	mitoxantrone	0.56	0.40	0.25	-12.12
692228	etoposide	0.71	0.31	0.32	-14.27
692736	mitoxantrone	0.57	0.37	0.28	-9.68
693167	amsacrine	0.54	0.35	0.16	-8.11
693169	etoposide	0.57	0.26	0.26	-10.45
693325	amsacrine	0.60	0.36	0.06	-5.46
693335	amsacrine	0.57	0.40	0.17	-6.88
693539	etoposide	0.52	0.38	0.33	-7.72
693543	mitoxantrone	0.57	0.48	0.23	-12.99
693546	etoposide	0.53	0.44	0.30	-9.28
693563	amsacrine	0.58	0.31	0.26	-9.25
693858	amsacrine	0.59	0.40	0.20	-9.06
694456	mitoxantrone	0.54	0.34	0.26	-10.17
694501	mitoxantrone	0.61	0.30	0.30	-7.65
695590	mitoxantrone	0.56	0.05	0.30	-11.22
695804	etoposide	0.54	0.34	0.22	-9.17
695936	etoposide	0.56	0.33	0.25	-13.53
696125	amsacrine	0.62	0.39	0.27	-9.10
697535	amsacrine	0.58	0.35	0.25	-9.81
697653	amsacrine	0.55	0.41	0.22	-8.18
697685	mitoxantrone	0.54	0.31	0.12	-8.61
697686	etoposide	0.71	0.28	0.25	-7.63
697726	mitoxantrone	0.53	0.37	0.15	-7.89
698104	amsacrine	0.55	0.42	0.24	-10.94
699087	etoposide	0.54	0.40	0.31	-9.48
699150	mitoxantrone	0.57	0.43	0.27	-11.06
699724	mitoxantrone	0.55	0.48	0.23	-10.86
701022	etoposide	0.58	0.34	0.26	-8.89
701029	etoposide	0.57	0.39	0.18	-10.30
701599	mitoxantrone	0.54	0.29	0.19	-8.65
701616	ametantrone	0.65	0.46	0.23	-11.20
701660	etoposide	0.60	0.35	0.27	-8.38
701743	mitoxantrone	0.58	0.05	0.27	-10.64
701745	ametantrone	0.56	0.39	0.24	-11.63
701747	etoposide	0.64	0.36	0.26	-11.33
701973	mitoxantrone	0.68	0.33	0.20	-12.81
702045	etoposide	0.54	0.33	0.25	-8.71
702047	etoposide	0.55	0.37	0.17	-8.13
703104	amsacrine	0.61	0.41	0.21	-7.54
703327	etoposide	0.61	0.37	0.19	-11.59
703445	etoposide	0.53	0.37	0.23	-9.81
703874	etoposide	0.57	0.34	0.24	-7.79
704533	mitoxantrone	0.54	0.33	0.18	-8.02

Continued on next page

NSC	lead	BiolAct	ROCS	CFP	docking
706000	ametantrone	0.59	0.29	0.22	-12.61
706003	mitoxantrone	0.53	0.28	0.22	-12.51
706737	etoposide	0.54	0.30	0.34	-7.89
706743	mitoxantrone	0.54	0.48	0.33	-12.92
706744	amsacrine	0.74	0.40	0.24	-12.92
707059	etoposide	0.56	0.37	0.25	-7.91
707068	etoposide	0.54	0.36	0.21	-5.61
707696	etoposide	0.53	0.35	0.31	-8.61
708061	etoposide	0.57	0.20	0.12	-6.96
708411	etoposide	0.52	0.31	0.20	-6.63
708417	amsacrine	0.53	0.31	0.20	-7.52
708446	etoposide	0.57	0.35	0.26	-8.74
708447	mitoxantrone	0.57	0.18	0.20	-9.99
709317	etoposide	0.68	0.44	0.34	-7.33
709358	mitoxantrone	0.54	0.30	0.29	-9.52
710112	etoposide	0.54	0.33	0.29	-10.12
710403	etoposide	0.60	0.32	0.31	-8.77
710525	etoposide	0.60	0.34	0.26	-8.60
710551	ametantrone	0.59	0.34	0.24	-13.52
710578	etoposide	0.55	0.31	0.27	-8.22
710770	etoposide	0.62	0.35	0.28	-8.27
710861	etoposide	0.57	0.31	0.19	-8.68
711312	etoposide	0.54	0.26	0.21	-7.95
711693	ametantrone	0.53	0.26	0.28	-11.73
711694	ametantrone	0.56	0.26	0.28	-12.00
711695	mitoxantrone	0.54	0.29	0.32	-12.23
711696	ametantrone	0.65	0.24	0.28	-12.31
711946	mitoxantrone	0.58	0.27	0.32	-12.63
712583	mitoxantrone	0.62	0.18	0.03	-5.46
712741	amsacrine	0.53	0.45	0.25	-9.58
713309	etoposide	0.67	0.41	0.31	-9.02
713649	etoposide	0.66	0.34	0.27	-8.18
713663	etoposide	0.57	0.34	0.33	-7.56
713672	mitoxantrone	0.55	0.32	0.26	-10.00
715974	etoposide	0.61	0.31	0.16	-7.93
716095	etoposide	0.53	0.36	0.28	-7.50
718163	etoposide	0.54	0.39	0.21	-7.78
718303	etoposide	0.54	0.32	0.30	-8.52
718630	mitoxantrone	0.68	0.37	0.26	-7.62
718631	etoposide	0.69	0.33	0.24	-7.91
718810	etoposide	0.60	0.30	0.18	-6.82
720538	mitoxantrone	0.59	0.30	0.20	-9.00
720555	etoposide	0.53	0.37	0.22	-10.31
720597	etoposide	0.56	0.32	0.17	-8.93
720689	ametantrone	0.63	0.37	0.28	-14.43
720861	etoposide	0.60	0.35	0.23	-9.40
721385	mitoxantrone	0.56	0.34	0.20	-8.53
721386	amsacrine	0.58	0.35	0.18	-9.83
721387	mitoxantrone	0.64	0.37	0.23	-11.06
721514	mitoxantrone	0.59	0.39	0.25	-8.87
721685	etoposide	0.53	0.35	0.24	-8.90
722900	mitoxantrone	0.69	0.40	0.31	-9.62
723387	amsacrine	0.74	0.42	0.24	-11.13
723554	etoposide	0.53	0.32	0.31	-9.55
723733	etoposide	0.67	0.28	0.32	-9.58
724562	amsacrine	0.53	0.39	0.24	-11.29
724563	mitoxantrone	0.54	0.45	0.32	-12.68
724995	amsacrine	0.61	0.39	0.24	-10.84
724998	mitoxantrone	0.59	0.45	0.32	-10.59
725026	amsacrine	0.55	0.38	0.18	-7.06
725665	etoposide	0.59	0.38	0.33	-10.41
725666	amsacrine	0.63	0.39	0.24	-10.54
725669	etoposide	0.54	0.31	0.24	-11.58
725671	amsacrine	0.66	0.40	0.21	-11.94
725775	amsacrine	0.66	0.40	0.24	-10.96
725776	amsacrine	0.53	0.40	0.24	-11.58
725981	etoposide	0.66	0.33	0.34	-9.12
726112	etoposide	0.57	0.39	0.34	-11.32
726331	amsacrine	0.54	0.38	0.25	-11.00
726332	amsacrine	0.59	0.43	0.24	-10.22

Continued on next page

NSC	lead	BiolAct	ROCS	CFP	docking
726627	etoposide	0.52	0.33	0.24	-9.31
726764	ametantrone	0.55	0.38	0.24	-13.06
726769	mitoxantrone	0.64	0.48	0.29	-11.98
726770	amsacrine	0.67	0.40	0.20	-10.48
726796	etoposide	0.56	0.30	0.22	-8.47
726863	etoposide	0.59	0.28	0.15	-6.30
726971	amsacrine	0.66	0.40	0.24	-11.12
727212	etoposide	0.53	0.36	0.33	-13.05
727354	mitoxantrone	0.61	0.32	0.31	-10.63
727356	mitoxantrone	0.63	0.44	0.32	-11.56
727572	etoposide	0.63	0.36	0.32	-8.06
727620	mitoxantrone	0.62	0.06	0.32	-14.04
727621	etoposide	0.53	0.38	0.33	-11.37
727722	mitoxantrone	0.58	0.39	0.30	-10.97
727729	mitoxantrone	0.70	0.30	0.28	-9.76
728086	etoposide	0.55	0.29	0.24	-8.39
728089	mitoxantrone	0.69	0.44	0.33	-12.15
728091	mitoxantrone	0.63	0.42	0.32	-9.88
728134	etoposide	0.66	0.36	0.26	-8.31
728219	etoposide	0.60	0.32	0.17	-7.54
728316	mitoxantrone	0.76	0.43	0.32	-10.79
728318	mitoxantrone	0.55	0.46	0.33	-12.16
728457	etoposide	0.60	0.26	0.22	-8.62
728594	etoposide	0.58	0.27	0.13	-7.54
728856	mitoxantrone	0.54	0.45	0.28	-13.04
728979	amsacrine	0.57	0.40	0.23	-9.37
729352	mitoxantrone	0.55	0.34	0.31	-10.86
729812	amsacrine	0.66	0.40	0.23	-9.54
729813	amsacrine	0.66	0.38	0.23	-11.23
730001	etoposide	0.53	0.32	0.26	-10.15
730435	ametantrone	0.54	0.42	0.24	-12.87
731155	mitoxantrone	0.53	0.44	0.30	-11.26
731981	amsacrine	0.60	0.41	0.18	-8.51
732534	mitoxantrone	0.55	0.18	0.21	-4.48
732693	etoposide	0.56	0.37	0.23	-8.14
732787	etoposide	0.61	0.36	0.29	-9.12
732836	etoposide	0.65	0.35	0.23	-8.97
732851	etoposide	0.52	0.35	0.25	-8.58
733465	etoposide	0.62	0.38	0.25	-8.54
733654	amsacrine	0.70	0.40	0.23	-9.86
733883	mitoxantrone	0.56	0.39	0.32	-9.70
734294	amsacrine	0.64	0.41	0.20	-9.39
734296	etoposide	0.65	0.37	0.32	-9.12
734430	amsacrine	0.58	0.34	0.17	-10.29
734561	etoposide	0.53	0.34	0.29	-10.91
734562	etoposide	0.66	0.34	0.29	-11.06
734799	amsacrine	0.58	0.41	0.21	-9.74
735004	etoposide	0.55	0.27	0.10	-8.31
735036	amsacrine	0.72	0.40	0.24	-12.35
735037	mitoxantrone	0.71	0.42	0.32	-10.00
735356	etoposide	0.56	0.42	0.22	-8.28
735404	etoposide	0.56	0.29	0.22	-9.60
735493	amsacrine	0.61	0.43	0.25	-11.53
735798	ametantrone	0.58	0.26	0.24	-12.27
735799	etoposide	0.57	0.31	0.34	-10.47
736101	etoposide	0.55	0.37	0.24	-9.75
736116	mitoxantrone	0.55	0.43	0.31	-11.46
736198	mitoxantrone	0.67	0.41	0.29	-9.50
736200	mitoxantrone	0.68	0.41	0.29	-9.67
736201	mitoxantrone	0.60	0.43	0.29	-9.77
736202	mitoxantrone	0.64	0.42	0.29	-9.09
736268	etoposide	0.53	0.33	0.30	-7.46
736394	amsacrine	0.64	0.41	0.21	-11.68
736493	etoposide	0.58	0.34	0.27	-10.72
736611	mitoxantrone	0.63	0.49	0.19	-12.61
736624	mitoxantrone	0.61	0.43	0.29	-9.66
736681	etoposide	0.63	0.37	0.29	-9.89
736829	mitoxantrone	0.55	0.48	0.29	-11.77
736913	etoposide	0.61	0.36	0.29	-9.91
736916	etoposide	0.65	0.36	0.29	-9.62

Continued on next page

NSC	lead	BiolAct	ROCS	CFP	docking
736988	amsacrine	0.65	0.43	0.25	-11.47
737318	mitoxantrone	0.56	0.41	0.32	-10.13
737344	amsacrine	0.65	0.37	0.24	-11.28
737518	amsacrine	0.70	0.40	0.24	-10.47
737672	mitoxantrone	0.58	0.43	0.34	-8.86
737707	amsacrine	0.54	0.41	0.20	-7.78
737737	mitoxantrone	0.65	0.36	0.24	-8.78
740051	etoposide	0.57	0.41	0.31	-9.06
740121	etoposide	0.58	0.30	0.21	-10.44
740268	mitoxantrone	0.64	0.41	0.33	-9.35
740504	etoposide	0.56	0.36	0.27	-13.06
740520	mitoxantrone	0.63	0.40	0.30	-9.98
740524	amsacrine	0.60	0.41	0.24	-10.16
740564	mitoxantrone	0.58	0.44	0.30	-11.47
740609	etoposide	0.52	0.34	0.28	-11.36
740643	amsacrine	0.63	0.42	0.24	-9.81
740644	amsacrine	0.65	0.40	0.24	-9.85
740645	amsacrine	0.66	0.40	0.24	-11.14
740648	amsacrine	0.67	0.46	0.25	-12.30
740974	mitoxantrone	0.60	0.43	0.30	-9.88
740975	mitoxantrone	0.59	0.41	0.29	-9.84
741106	amsacrine	0.60	0.43	0.20	-11.26
741107	amsacrine	0.57	0.40	0.22	-11.31
741459	mitoxantrone	0.76	0.30	0.28	-9.12
741695	etoposide	0.59	0.25	0.22	-8.64
742417	etoposide	0.57	0.30	0.27	-8.19
742801	etoposide	0.58	0.38	0.23	-7.78
743864	mitoxantrone	0.54	0.33	0.28	-8.88
744330	mitoxantrone	0.66	0.26	0.28	-8.80
744622	amsacrine	0.53	0.27	0.17	-9.49
746351	amsacrine	0.58	0.41	0.18	-7.28
747292	amsacrine	0.53	0.34	0.21	-8.17
751393	etoposide	0.61	0.37	0.27	-7.71

Table S7: Docking scores of DUDE-E decoys.

Title	docking_score
C65469915	-12.20
C00636200	-11.64
C02582595	-11.14
C14992360	-11.09
C57773689	-11.05
C20132031	-10.84
C15721960	-10.83
C76971785	-10.74
C09239600	-10.67
C97334294	-10.63
C71788398	-10.57
C98178696	-10.49
C39500252	-10.45
C05980708	-10.43
C38517546	-10.41
C19229474	-10.40
C02348787	-10.36
C63698417	-10.33
C63855242	-10.23
C15722286	-10.23
C33697219	-10.23
C08076202	-10.20
C64158213	-10.17
C02437529	-10.16
C38999931	-10.16
C13539040	-10.15
C64067023	-10.15
C34250406	-10.13
C65405674	-10.12
C40801473	-10.07

Continued on next page

Title	docking_score
C39560948	-10.05
C77357347	-10.02
C20116877	-9.99
C40570282	-9.99
C65407120	-9.97
C00636047	-9.95
C13539043	-9.93
C38486049	-9.91
C38517641	-9.90
C39015840	-9.89
C39015836	-9.88
C72434813	-9.86
C26896594	-9.80
C33450962	-9.79
C43773452	-9.79
C25585272	-9.79
C72477679	-9.77
C06510920	-9.76
C08740221	-9.74
C39953335	-9.74
C38546965	-9.73
C14472334	-9.71
C34001737	-9.70
C03840128	-9.68
C01698989	-9.65
C72380005	-9.65
C95869711	-9.63
C01062118	-9.57
C96336260	-9.55
C14086129	-9.52
C21146606	-9.52
C14744245	-9.52
C43773453	-9.50
C25605891	-9.45
C09490572	-9.42
C73374099	-9.42
C06614498	-9.40
C19741840	-9.38
C39932294	-9.38
C93791735	-9.37
C39946867	-9.37
C49061297	-9.37
C15066260	-9.36
C44358916	-9.34
C63818501	-9.29
C20805442	-9.29
C22576466	-9.28
C64683770	-9.27
C57761945	-9.27
C20463116	-9.22
C20436792	-9.22
C97945339	-9.20
C67616676	-9.18
C89943041	-9.18
C34914020	-9.18
C42182418	-9.14
C65374419	-9.14
C89943040	-9.14
C20915747	-9.12
C92455417	-9.12
C00623661	-9.12
C13555666	-9.12
C06501754	-9.11
C21866363	-9.10
C04066639	-9.09
C63503255	-9.09
C12504524	-9.08
C05192427	-9.08
C20836850	-9.05
C40756782	-9.05

Continued on next page

Title	docking_score
C40801537	-9.00
C04221602	-9.00
C14476043	-8.95
C01355376	-8.95
C97405180	-8.94
C40701948	-8.94
C58379195	-8.93
C49421241	-8.91
C40701949	-8.90
C86066393	-8.89
C88943634	-8.88
C08613448	-8.88
C67634894	-8.88
C02429161	-8.87
C72099787	-8.84
C18158775	-8.82
C09003806	-8.82
C39952927	-8.80
C88757913	-8.79
C03009215	-8.77
C15772708	-8.75
C01325000	-8.73
C12134269	-8.73
C06161920	-8.71
C08636781	-8.68
C20922520	-8.68
C04827638	-8.67
C09242812	-8.66
C20922598	-8.64
C20550368	-8.61
C08643875	-8.60
C22839320	-8.57
C39007764	-8.56
C14276867	-8.56
C97527879	-8.56
C37866073	-8.56
C11661801	-8.56
C20921837	-8.55
C22056694	-8.53
C02474720	-8.52
C08152480	-8.50
C35981790	-8.49
C02584814	-8.49
C15962384	-8.48
C97648044	-8.47
C63426950	-8.44
C50440563	-8.44
C05414593	-8.41
C63920236	-8.41
C03464393	-8.41
C12859983	-8.40
C54804688	-8.39
C89054088	-8.38
C09750214	-8.37
C89943835	-8.37
C16756749	-8.37
C04502846	-8.36
C39012857	-8.35
C97648048	-8.34
C14551099	-8.33
C08905668	-8.32
C06074140	-8.29
C03583996	-8.28
C85425716	-8.26
C04388372	-8.23
C19827453	-8.23
C09350731	-8.17
C41021336	-8.16
C27420460	-8.16
C16210468	-8.14

Continued on next page

Title	docking_score
C31157904	-8.14
C07742097	-8.11
C63752986	-8.08
C13608616	-8.05
C36127331	-8.03
C44443373	-8.00
C06550815	-7.98
C97337215	-7.95
C08763931	-7.94
C14674332	-7.88
C27570104	-7.87
C04221674	-7.86
C10248529	-7.84
C40679521	-7.83
C01754852	-7.83
C20566341	-7.83
C35555986	-7.83
C13608697	-7.80
C20566737	-7.80
C40734699	-7.77
C01037587	-7.76
C08740529	-7.75
C88464716	-7.74
C40686541	-7.71
C03807172	-7.68
C03043911	-7.66
C59612440	-7.65
C92477162	-7.61
C09325300	-7.60
C23379430	-7.59
C36006179	-7.59
C70671778	-7.59
C44137709	-7.56
C01511355	-7.55
C16283587	-7.55
C11883249	-7.51
C19940784	-7.50
C12930324	-7.46
C59147401	-7.46
C18098320	-7.43
C13513671	-7.40
C96147944	-7.38
C20922797	-7.38
C21819441	-7.38
C16430580	-7.36
C05362734	-7.34
C20407810	-7.33
C71789697	-7.32
C12516987	-7.31
C64684229	-7.30
C14609098	-7.30
C02751023	-7.29
C06560016	-7.25
C58441612	-7.25
C96395303	-7.21
C64948688	-7.09
C01452524	-7.08
C08116979	-6.98
C33257185	-6.89
C05819214	-6.85
C36694022	-6.76
C32996316	-6.75
C09366196	-6.68
C01820789	-6.66
C64684771	-6.66
C07260781	-6.61
C96333100	-6.52
C36693907	-6.51
C09824460	-6.51
C04742125	-6.22

Continued on next page

Title	docking_score
C98046891	-5.98
C29559740	-5.43
C03874159	-5.23
C16923900	-5.07
C67911450	-3.71
